# Experimental Annealing of Zircon: Influence of Inclusions on Stability, Intracrystalline Melt Migration, Common Lead Leaching, and Permeability to Fluids

**DOI:** 10.1021/acsearthspacechem.1c00212

**Published:** 2022-01-11

**Authors:** Irene Morales, José F. Molina, Aitor Cambeses, Pilar Montero, Fernando Bea

**Affiliations:** Departamento de Mineralogía y Petrología, Facultad de Ciencias, Campus de Fuentenueva, University of Granada, 18071 Granada, Spain

**Keywords:** zircon annealing, mineral inclusions, glass inclusions, melt migration, baddeleyite−zircon stability relationships, tungstate dissolution−reprecipitation, common Pb leaching

## Abstract

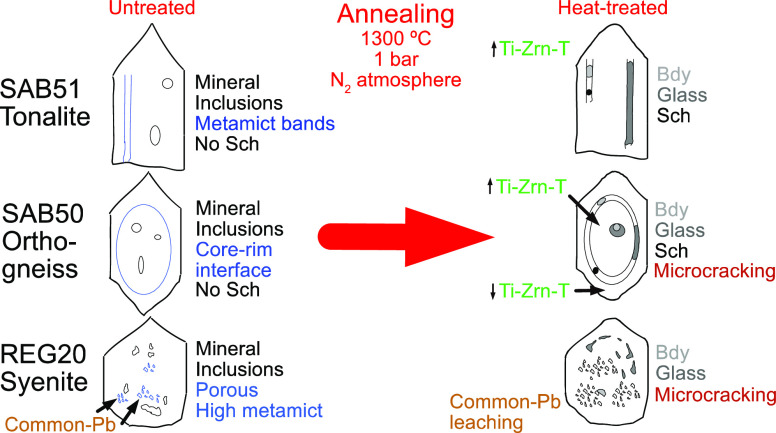

Zircon derived from crustal rocks can survive dissolution into hot basalts during magma hybridization and rock assimilation if it is shielded as an inclusion phase in early-formed phenocrysts or in minerals from non-disaggregated xenoliths. Under these conditions, zircon can be thermally shocked, triggering recrystallization of metamict domains and reaction with its hosted mineral inclusions. This work simulates this process by performing thermal annealing experiments on zircon grains with variable degrees of metamictization. These were embedded in cristobalite powder under a N_2_ atmosphere at 1 bar and 1300 °C. The thermal annealing produces recrystallization of metamict domains, melting of multi-phase mineral inclusions, nanopore formation, and microcrack propagation by thermo-elastic stress. The porosity enhances intracrystalline melt mobility, leaching out trace-element and mineral impurities. Baddeleyite was formed at temperatures below the thermal decomposition of pure zircon by two mechanisms: (i) recrystallization of metamict domains assisted by silica migration from the reaction site and (ii) incongruent zircon dissolution into molten mineral inclusions with a high CaO/SiO_2_ ratio. Highly metamict zircons with elevated common Pb and radiogenic Pb loss, which were impossible to date with SHRIMP, lost all their common Pb and some radiogenic Pb upon annealing, producing well-fitted discordias with a significant upper intercept age.

## 1 Introduction

Zircon is currently the most analyzed mineral by Earth scientists because it provides valuable information about the age and origin of all rock types. However, several aspects of zircon behavior and stability during geological processes are not yet adequately studied. The behavior of zircon during ultra-high-*T* metamorphism and entrainment in hot magmas stands out among such inadequately studied processes. If shielded from the melt to prevent dissolution, zircon grains can withstand temperatures much higher than those at which they crystallized and may remain stable. Trace-element diffusion along chemical gradients can occur under such conditions.^1^ However, if zircon grains have fusible inclusions and these melt, the so-formed liquids can react with the host zircons and migrate within the crystal, leaching out impurities, common Pb included. Previous annealing experiments have demonstrated the process, showing how molten mineral inclusions of feldspathic composition are efficient Pb sinks that cause Pb loss discordias.^2^

**Figure 1:**
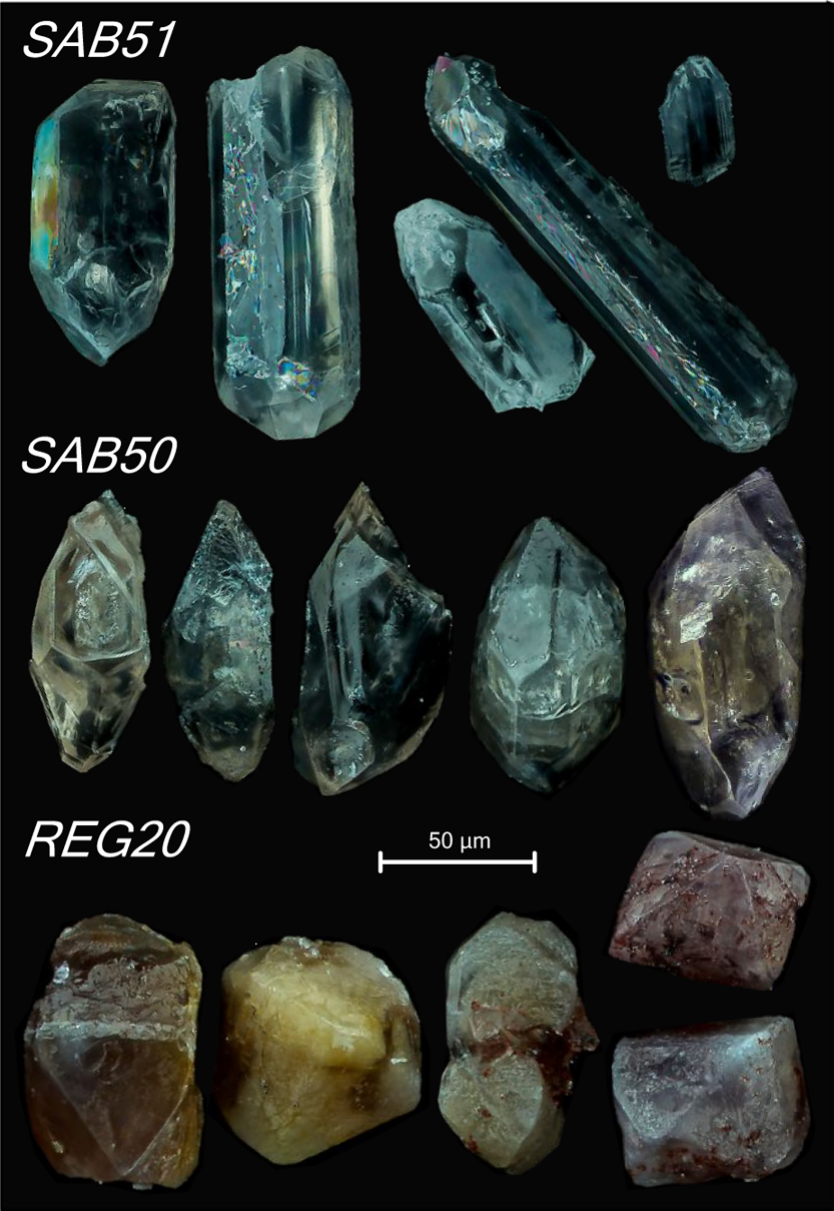
Macro-photographs of untreated zircon grains from tonalite SAB51, orthogneiss SAB50, and syenite REG20. Untreated SAB51 zircons are euhedral transparent and colorless to slightly pinkish, most of them forming long narrow prisms terminated by short pyramids. Untreated SAB50 zircons are transparent and colorless or slightly pinkish, mostly euhedral short stubby prisms terminated by long pyramids. Highly metamict REG20 zircons are euhedral to subhedral, translucent to opaque with different colors (from yellow to purple and brown), forming short stubby prisms terminated by short pyramids. Images obtained by optical microscopy using a focus stacking technique and non-polarized visible light (see Section 2.2. for details).

**Figure 2:**
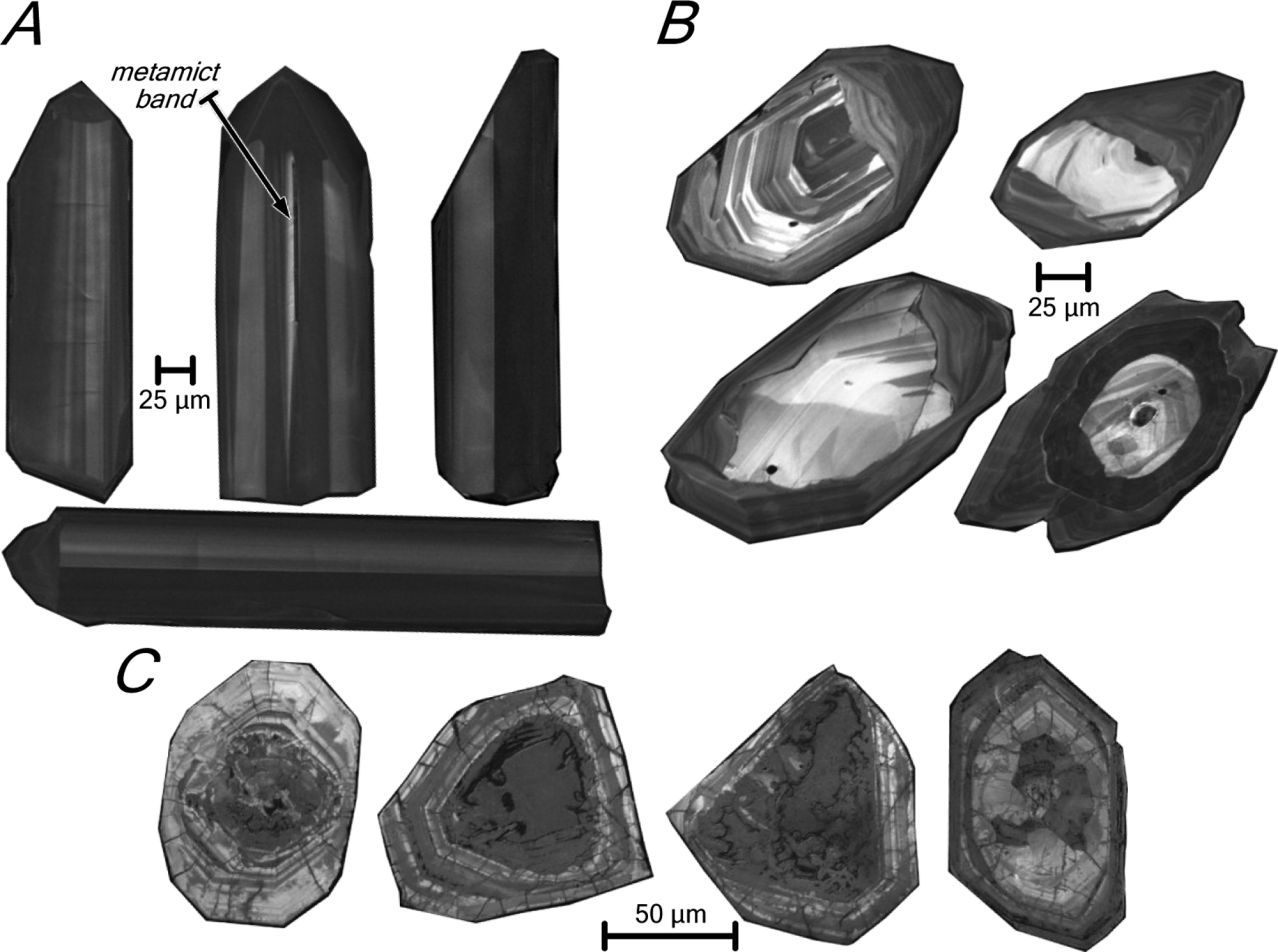
CL images of untreated zircon grains from tonalite SAB51, orthogneiss SAB50, and syenite REG20. (A) SAB51 zircons appear zoned with alternating light-gray and dark-gray bands parallel to the longest axis. (B) SAB50 zircons show oscillatory zoning and an Ediacaran euhedral inherited core overgrown by a Cambro–Ordovician rim, which generally truncates the core zoning. (C) Highly metamict REG20 zircons show a marked oscillatory zoning, locally partially obliterated, and abundant inclusions and cracks, in most cases with a radial disposition.

This work studies the textural and compositional variations experienced by experimentally annealed natural zircon grains from three samples representing a broad spectrum of situations (Figures 1 and 2). The first sample is a Carboniferous tonalite with zircons of remarkable constant isotopic composition but with localized metamict zones that may be prone to melting and decomposition. The second is a Cambrian–Ordovician S-type orthogneiss in which most zircons have an Ediacaran core that is oriented differently with respect to the rims; this effect may cause thermal decompaction upon heating and open up space for melt migration. The third is an Early Paleoproterozoic to Neoarchean syenite with highly metamict and inclusion-rich zircons that show elevated common Pb and a marked radiogenic Pb loss, thus being impossible to obtain their accurate U/Pb age with SHRIMP (see Section 5.4); accordingly, these zircons seem suitable for studying the effects of annealing and within-crystal melt production on the behavior of common Pb.

The samples were annealed to investigate: (i) the reactions between zircon and its inclusions; (ii) if these latter liquefy, how the melt that is produced migrates within the crystals; and (iii) whether melt migration may remove Pb and other trace elements.

## 2 Methods

### 2.1 Annealing Procedure

Annealing experiments were carried out in a horizontal tubular furnace in a N_2_ atmosphere using open cylindrical alumina crucibles with a diameter of 12 mm and height of 14 mm, as described in Bea et al.^2^ Experiments were conducted over 6 months at 1300 °C, which was enough to fuse most inclusions, enhance cation diffusion, and cause perceptible chemical and isotopic variations.^2^ At these temperatures, annealing experiments by Váczic et al.^3^ show that (both fresh and altered) natural and synthetic zircons heated in either alumina, zirconia, or platinum crucibles decomposed quickly (within 96 h) to baddeleyite plus silica. However, we have found in previous experimental work (see Bea et al.^2^) that embedding the zircons in silica stops the decomposition and permits temperatures of 1300–1450 °C to be sustained over several months. Accordingly, the experiments in this study were done by embedding the zircons in pure cristobalite prepared from fumed silica (see details in Bea et al.^2^).

### 2.2 Analytical Methods

Zircon concentrates were dried, hand-picked, and placed in a conductive carbon layer to study their morphology—first using a focus stacking technique under an optical microscope using a camera (Evil Sony A7RII) with Mitutoyo M PLAN APO microscope objectives and following that using a variable-pressure scanning electron microscope (ZEISS LEO 1430-VP) with secondary electron (SE) and BSE detectors. Then, the studied zircon grains were recovered, mounted in epoxy, polished, and documented with a high-vacuum scanning electron microscope (ZEISS EVO-10) equipped with SE, BSE, CL, and EDAX detectors. The grains were analyzed with EMP, LA-ICP-MS, and SHRIMP if deemed necessary by their mineralogical and textural features. All instrumental analyses were done in the Centro de Instrumentación Científica (CIC) of the University of Granada.

Identification of mineral and glass inclusions hosted in zircon grains and determination of their major-element composition were carried out by energy-dispersive X-ray (EDX) analysis.

The W concentrations were measured using a CAMECA SX-100 electron microprobe because in ICP–MS, the four tungsten isotopes, ^182^W, ^183^W, ^184^W, and ^186^W show interference with the abundant erbium oxides (^16^O^166^Er^+^, ^16^O^167^Er^+^, ^16^O^168^Er^+^, and ^16^O^170^Er^+^) that are produced during the ablation of zircon. We used metallic W as a standard. The Ti contents (measured on the isotope ^49^Ti, free from isobaric interferences) and the trace element compositions of zircon grains were determined using a Perkin Elmer NexION 350X ICP–MS system coupled to a New Wave Research NWR 213 laser. The NIST-610 glass, used as an external standard, was measured after every six unknowns. The analyzed spots were pre-ablated for 15 s with a laser fluency of 2.5 J cm^–2^ and then ablated for 60 s with a laser fluency of 7.5 J cm^–2^. A blank, measured using the same conditions but with zero laser energy, preceded every measurement. Data reduction was made using LA-ICP-MS software written in the STATA programming language (available from F. Bea upon reasonable request). Each analysis consisted of 60 mass scans. The counts for selected elements, P, Ti, La, Y, Hf, Pb, U, and Th, obtained in each mass scan were projected against the scan number to identify count spikes that might indicate tiny inclusions within the ablated zircon crater. If such spikes were seen, the whole analysis was discarded. This was the case for more than 100 zircon grains of the syenite REG20, in which the abundance of inclusions and cracks (Figures 1 and 2) prevented us from obtaining clean zircon analyses. Reducing the beam diameters does not help significantly because the laser pit was always about 60 μm (or more) deep.

In contrast, SHRIMP analyses of the REG20 zircons were possible because the ^16^O^16^O^+^ primary beam crater was about 1–2 μm deep. Therefore, we used this technique for U–Th–Pb isotope analyses, following the method described by Williams and Claesson,^4^ except counting times and peak centering that must be adapted to the age of zircons. The analyses were obtained using a SHRIMP IIe/mc ion microprobe at the IBERSIMS laboratory (CIC). Uranium concentration was calibrated using the SL13 reference zircon (U: 238 ppm;^5^). U/Pb ratios were calibrated using the TEMORA-II reference zircon (417 Ma;^6^). Isotope ratios and approximate ages generated with built-in PRAWN software were captured and further processed with the SHRIMPTOOLS software (downloadable from www.ugr.es/fbea) written with the STATA programming language.

## 3 Starting Zircons and Their Inclusions

We used natural zircon grains from the three zircon-rich rocks mentioned before. Two of these were described in detail by Bea et al.:^2^ (i) the tonalite SAB51 from Sanabria appinites in the Central Iberian Zone and (ii) the orthogneiss SAB50 from the Ollo de Sapo Formation in the same area. The other sample is the Neoarchean silica-saturated syenite REG20 from the Awsard kalsilite–nepheline syenitic complex in the Reguibat Shield of the Western African Craton, described in Bea et al.^7^ The inclusions found in the untreated zircon grains and their main textural features are summarized in Table 1. The major-element compositions of selected mineral inclusions are listed in Table 2, whereas trace-element compositions of the host zircons are reported in Tables 3 and 4.

**Table 1:** Summary of Mineral Assemblages and Textures in Untreated Zircon Grains^a^

sample	rock type	grain number	mineral inclusions	main textural features
SAB51	tonalite	194	Qz (1)	tear drop shapes
			Kfs (4)	
			Fl (1)	
			Qz + Ab + Kfs (2)	tear drop-shaped globules
			FS (1)	partially filled coarse gas bubbles
			AFM (1)	euhedral shapes
				
SAB50	orthogneiss	212	Qz (9)	tear drop and anhedral shapes
			Kfs (6)	
			Chl (2)	
			Ap and F-Ap (20)	
			Rt (1)	
			Hc (1)	
			Fl (5)	
			FS (1)	
			AFM (1)	
			Qz + Afs (1)	tear drop-shaped globules
			Qz + Kfs + Ilm (1)	
			Pl + Bt + Ilm (1)	
			Qz + Mnz + F-Ap (1)	
				
REG20	syenite	133	F-Ap (6)	anhedral shapes
			Thr/Hut (4)	
			Che–Hut (4)	
			Bt + Ab + Kfs (1)	
			Ab + Mag + Ep + Aln (1)	
			Kfs + Mag (1)	
			secondary phyllosilicates and Ep (5)	tiny grains infilling microcracks

a Mineral abbreviations after Whitney and Evans.^8^ Other abbreviations: AFM = aluminofluoride mineral (probably hydrokenoralstonite); Che–Hut = cheralite–huttonite solid solution; FS = fluorosilicate; and Thr/Hut = thorite/huttonite. The number of grains with the reported inclusions indicated in parentheses.

**Table 2:** Selected EDX Analyses of Mineral Inclusions (Atoms per Formula Unit)

sample	SAB51				SAB50				REG20				
rock type	tonalite				orthogneiss				syenite				
type^a^	U	U	A	A	U	U	U	A	U	U	U	U	U
mineral^b^	AFM	MB Zrn	Sch	Bdy	AFM	F-Ap	FS	Sri	F-Ap	Thr/Hut	Kfs	Pl	Che–Hut
point	4	133	32	199	3	82	7	125	57	6	5	26	40
no of atoms	2.5 Cat	4 O	4 O	1 Cat	2.5 Cat	12.5 O	11 O	3 Cat	12.5 O	4 O	5 Cat	5 Cat	4 Cat
Si		1.556					3.488			0.944	2.952	2.867	1.194
Ti								2.559					
Al	0.914				1.007		2.172	0.034			1.015	0.99	
Fe	0.142				0.108	0.075	0.158					0.171	
Mn					0.014								
Mg	0.896	0.066			0.868		0.251						
Ca	0.547	0.181	0.911		0.111	4.898			5.01	0.119		0.107	0.633
Na					0.392						0.039	0.865	
K		0.045					0.715				0.993		
F	5.645	4.986			5.62	1.097	2.585		1.235	0.525			
Cl	0.034				0.024								
P						3.102			2.99				0.611
W			1.03										
Zr		2.152		0.986				0.407					
Hf				0.014									
Th										0.937			1.562
total	8.18	8.986	1.941	1	8.144	9.172	9.369	3	9.235	2.525	5	5	4

a A = annealed; U = untreated.

b Mineral abbreviations after Whitney and Evans.^8^ Other abbreviations: AFM = aluminofluoride mineral (probably hydrokenoralstonite); Che–Hut = cheralite–huttonite solid solution; FS = fluorosilicate; Thr/Hut = thorite/huttonite; MB = metamict band; and Sri = srilankite.

**Table 3:** LA-ICP-MS Analyses of Trace Elements in Zircon Grains (Data in ppm)

sample	SAB51, tonalite													
type	untreated							annealed						
point	1	2	3	5	50	56	57	4	10	15	48	53	54	55
texture^a^	LB	LB	LB	LB	DB	DB	DB	LB	LB	LB	DB	DB	DB	DB
P	638	659	408	321	299	663	392	203	310	130	223	346	325	241
Ti	10.3	8.11	16.0	13.6	8.02	7.69	9.98	29.4	21.5	32.2	6.94	34.7	11.7	41.0
Y	1015	974	1164	1033	1385	1664	1487	1793	718	524	1064	906	1307	978
La	0.67	0.7	0.63	0.8	0.70	2.80	0.60	0.40	0.28	0.08	0.44	1.61	0.83	1.16
Ce	6.87	6.41	7.09	7.29	7.93	11.2	8.04	6.82	3.90	4.37	7.31	6.38	5.71	3.47
Pr	0.73	0.76	0.77	0.69	0.89	1.36	1.07	0.40	0.20	0.58	0.58	1.02	0.93	0.13
Nd	7.73	9.19	8.42	7.01	11.0	13.8	12.4	13.2	3.81	6.24	3.89	6.01	18.2	4.42
Sm	10.1	11.7	12.1	8.86	18.3	17.9	18.3	27.9	11.8	3.55	16.7	6.38	16.1	9.18
Eu	1.67	1.91	1.88	1.52	2.61	2.94	3.08	3.34	2.67	0.62	1.00	0.42	3.83	0.59
Gd	39.8	43.6	47.1	33.6	65.9	70.7	65.7	78.5	34.9	7.79	42.7	42.0	60.6	40.9
Tb	11.6	12.7	13.5	10.6	17.8	21.3	18.7	17.6	12.9	6.23	13.8	11.5	18.0	12.1
Dy	122	123	145	114	181	211	190	231	79	46.2	108	101	139	107
Ho	40.1	41.1	47.7	38.3	57.1	71.6	60.7	61.8	29.4	20.9	39.9	29.8	45.0	33.8
Er	169	171	198	170	224	289	240	246	113	80.5	161	134	204	139
Tm	33.8	32.5	39.8	35	43.9	54.1	46.6	52.6	18.1	13.2	29.9	23.9	39.3	28.7
Yb	272	263	319	289	333	442	374	384	158	134	243	173	305	236
Lu	50.4	45.6	58	57	60.6	78.1	67.3	72.1	24.7	27.4	54.8	46.9	45.4	49.5
Hf	8477	7973	9106	8610	7647	7786	7977	8262	7554	8702	9913	7809	7142	8807
Pb	23	23.6	28.7	25.2	33.5	31.8	28.2	33.5	8.40	10.8	19.4	11.4	14.5	21.6
Th	213	225	251	191	352	428	385	430	121	101	227	186	244	202
U	377	349	440	404	481	522	464	519	194	191	334	235	306	340
Sc	240	255	252	256	283	266	256	na	na	na	na	na	na	na
Nb	0.48	0.52	0.54	0.56	0.80	0.71	0.73	na	na	na	na	na	na	na
Ta	0.38	0.37	0.43	0.33	0.43	0.44	0.46	na	na	na	na	na	na	na

sample	SAB50, orthogneiss													
type	untreated							annealed						
point	71	72	73	74	19	20	23	75	76	82	84	21	22	28
texture^a^	R	R	R	R	C	C	C	R	R	R	R	C	C	C
P	1460	989	860	860	877	888	712	1593	1296	1161	1666	853	387	1618
Ti	43.4	12.0	25.0	25.0	8.38	11.5	11.1	25.7	12.2	14.1	38.0	132	52.1	45.4
Y	3163	2403	2196	2196	2044	2214	1566	2138	3261	2963	3810	2155	1031	3076
La	0.18	0.15	0.22	0.20	0.25	0.32	0.32	0.16	0.04	0.27	1.26	1.00	0.16	0.07
Ce	3.84	2.39	2.30	2.30	3.15	7.21	10.1	3.53	0.36	1.58	3.93	27.4	11.5	8.58
Pr	0.19	0.17	0.11	0.22	0.20	0.54	0.23	0.32	0.26	0.27	0.84	1.45	0.21	0.46
Nd	2.63	1.58	1.72	1.72	2.07	4.94	3.63	3.70	4.63	5.37	8.01	5.66	2.98	3.62
Sm	5.16	3.91	3.24	3.24	2.76	4.63	4.39	3.97	3.59	5.96	7.76	6.08	2.93	2.15
Eu	0.24	0.43	0.18	0.18	0.11	0.42	0.60	0.77	0.62	1.18	1.99	1.25	0.25	0.62
Gd	35.6	26.4	22.6	22.6	20.8	24.4	23.2	16.8	36.9	39.0	29.7	42.1	29.7	22.3
Tb	16.4	12.8	12.1	12.1	11.1	12.1	10.1	12.4	17.2	17.6	17.2	16.5	10.5	12.9
Dy	247	188	184	184	169	183	136	183	268	219	230	186	99.7	263
Ho	112	83.3	77.9	77.9	76.7	79.6	57.4	68.4	112	97.9	97.3	71.1	34.9	93.9
Er	571	434	395	395	416	417	288	407	548	534	667	373	178	543
Tm	132	96.9	85.7	85.7	93.1	98.6	67.7	92.2	121	120	134	81.6	33.7	121
Yb	1118	848	723	723	768	873	596	818	1052	1149	1253	706	288	1153
Lu	239	177	148	148	153	174	125	167	213	214	272	143	61.9	211
Hf	9256	9123	9375	9375	8815	9281	8805	10 046	9832	9966	9021	7938	7306	9760
Pb	25.5	20.7	15.5	15.5	21.0	25.9	21.9	19.0	18.0	14.9	18.6	21.7	13.9	22.3
Th	44.4	29.6	19.3	19.3	19.2	48.1	46.0	20.7	30.4	20.7	19.4	139	43.9	25.5
U	294	219	162	162	212	260	185	193	221	194	210	163	102	243
Sc	449	379	460	454	290	388	438	na	na	na	na	na	na	na
Nb	0.73	0.43	0.39	bdl	0.28	0.71	1.08	na	na	na	na	na	na	na
Ta	bdl	bdl	Bdl	bdl	bdl	bdl	bdl	na	na	na	na	na	na	na

a LB = light band; DB = dark band; R = rim; C = core; bdl = below detection limit; and na = not analyzed.

**Table 4:** EMP Analyses of Mg, Ca, Fe, and W in Zircon Grains (Data in ppm)

sample	SAB51, tonalite														
type	untreated														
point	1	2	3	4	5	6	7	8	9	10	11	12	13	14	15
			H	H		H		H	H	H	H	H	H		H
texture^a^	MB	MB	LB	DB	MB	LB	MB	DB	LB	DB	LB	LB	DB	MB	DB
Mg	351	271	37	127	103	36	64	47	40	35	36	38	144	428	45
Ca	3945	3464		1000	1128		933	42					416	1791	19
Fe	41	43		25			58						45	100	
W	332	363		179	243		275	149	116	165	78	100	109	185	113

sample	SAB51, tonalite										
type	annealed										
point	1	2	3	4	5	6	7	8	12	13	14
	H		H	H	H	H		H	H	H	H
texture^a^	DB	RB	DB	LB	DB	LB	RB	LB	DB	LB	LB
Mg	92	95	62	55	148	23	442	40	41	36	37
Ca	138	184	37	36	349		2526				
Fe							39				
W	392	326	271	145	347	255	554	138	218	193	194

sample	SAB50, orthogneiss														
type	untreated														
point	1	2	3	4	5	6	7	8	9	10	11	12	13	14	15
texture^a^	R	C	R	C	R	R	R	R	C	C	C	C	R	R	R
Mg	38	33	34	38	39	38	36	36	38	42	38	36	114	38	58
Ca				399						2102	28	57	750		168
Fe				189			29		104	65		25	93		
W	437	199	256		99	654	477	554	218	186		126	1028	633	726

sample	SAB50, orthogneiss										
type	annealed										
point	1	2	3	4	5	6	7	8	12	13	14
texture^a^	R	C	R	C	R	C	R	C	C	C	R
Mg	31	36	33	34	38	41	41	83	214	39	37
Ca				18				62	316		
Fe											
W	590	195	601	124	369	200	200	188	220	267	476

a H = host; LB = light band; DB = dark band; MB = metamict band; RB = recrystallized band; R = rim; and C = core.

### Tonalite SAB51

**Figure 3:**
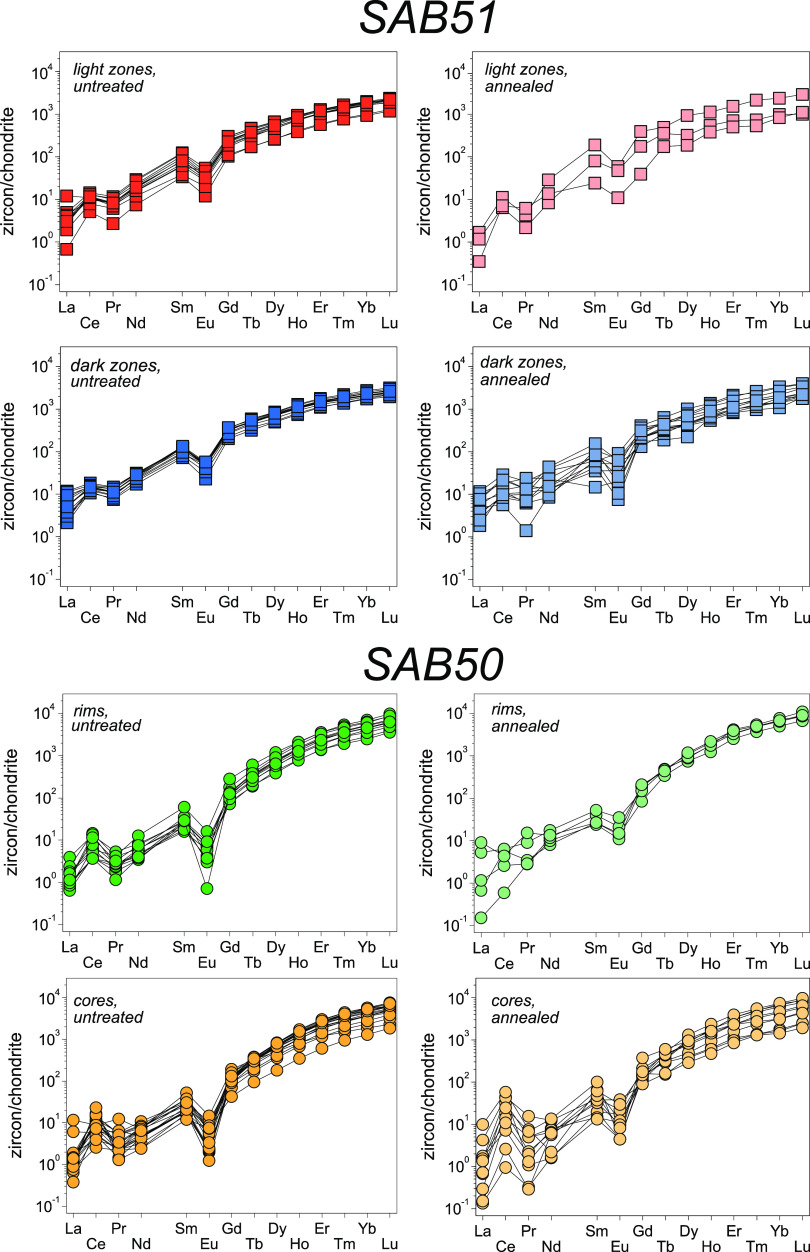
Chondrite-normalized REE patterns from untreated and annealed zircon grains from tonalite SAB51 and orthogneiss SAB50. SAB51: the darker bands are richer in HREE than the lighter ones. SAB50: the REE compositions of the rims and cores are clearly close to each other.

**Figure 4:**
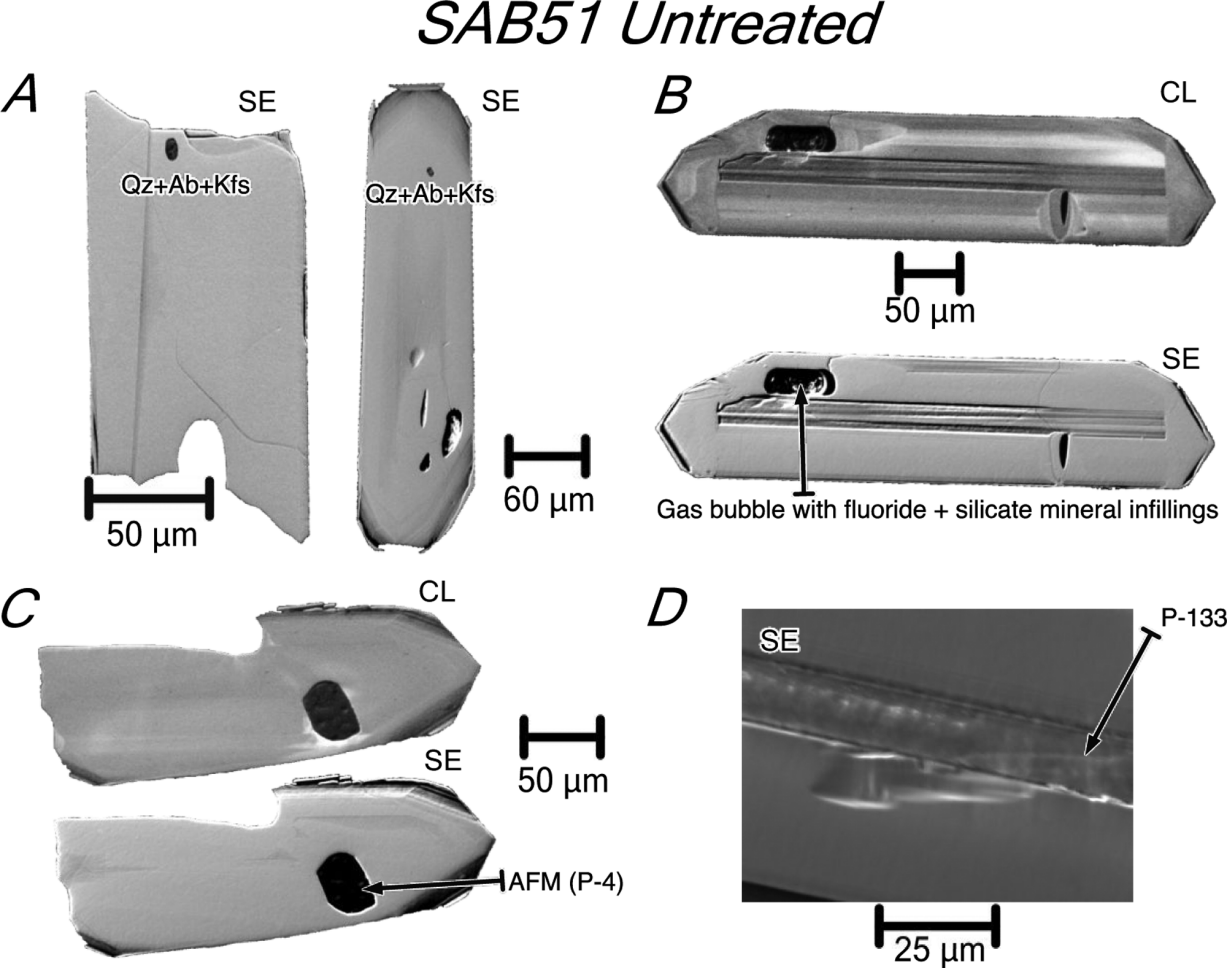
CL and SE images of untreated zircons from tonalite SAB51. The zircons present a slightly oscillatory zoning and non-luminescent metamict porous bands parallel to pyramidal and/or prismatic faces. (A) Zircons with metamict porous bands and polymineralic tear drop-shaped inclusions of quartz + albite + K-feldspar. (B) Zircon with non-luminescent metamict porous bands and a coarse gas bubble filled with fluoride + silicate minerals. (C) Zircon with non-luminescent metamict porous bands and euhedral inclusion of a complex Mg–Fe–Ca–Na aluminofluoride mineral (probably hydrokenoralstonite; see analysis in Table 2; cf.,^9^). (D) SE image of zircon with metamict porous bands containing impurities of Mg, Ca, K, and F (see analysis in Table 2). Mineral abbreviations after Whitney and Evans.^8^ Other abbreviations: AFM = aluminofluoride mineral.

This is an undeformed amphibole-biotite tonalite that contains abundant zircon with a concordant U–Pb age of 318 ± 2 Ma. Zircon grains have very little or no common Pb. They are euhedral, transparent, and colorless to slightly pinkish, most of them forming long (100–300 μm) narrow prisms terminated by short pyramids (Figure 1). Under cathodoluminescence (CL), most of them appear zoned with alternating light-gray and dark-gray bands parallel to the longest axis (Figure 2A). LA-ICP-MS analyses reveal that the darker bands are richer in U, Th, Y, and HREE than the lighter ones (Figure 3; Tables 3 and S1 from the Supporting Information). Some dark bands that contain detectable F and K are partially metamict and microporous (Figure 4D; Table 2). EMPA analyses of SAB51 zircons also show systematic variations in W abundances (Table 4) that are lower in the light bands [av. 99 ppm; range: from <dl (detection limit = 78 ppm) to 116 ppm] than in the darker ones (av. 138 ppm; range: 109–179 ppm), whereas in the metamict bands, they are higher with an average concentration of ca. 280 ppm (range: 185–363 ppm).

SAB51 tonalite zircons contain abundant monomineralic and polymineralic inclusions (Figure 4; Table 1). The former are made up of quartz, K-feldspar, fluorite, and a complex euhedral Mg–Fe–Ca–Al fluoride mineral (ca. 20–35 μm long), most probably hydrokenoralstonite (Figure 4C). The polymineralic inclusions consist of tear drop-shaped globules (ca. 5–20 μm long) of quartz + albite + K-feldspar (Figure 4A) that can be accompanied by relatively coarse gas bubbles (ca. 40 μm long), which are partially filled with fluoride + silicate mineral phases (Figure 4B).

### Orthogneiss SAB50

This is a Cambrian–Ordovician granitic orthogneiss that hosts the Variscan SAB51 tonalite. The orthogneiss is derived from a mildly deformed porphyritic peraluminous granite. It has a marked augen structure, with large crystals of K-feldspar between a coarse-grained and foliated groundmass. Despite deformation, microgranular enclaves and aplite dikes are still recognizable. The orthogneiss SAB50 is weakly metamorphosed, far below the grade of anatexis. It contains abundant zircon grains consisting of transparent, colorless, or slightly pinkish, mostly euhedral short stubby prisms (100–230 μm long) that are terminated by long pyramids (Figure 1). About 90–95% of them have an Ediacaran core (600–610 Ma) surrounded by a Cambrian–Ordovician rim (480–485 Ma) with zoning that truncates that of the core (Figure 2B)^10,11^ and shows a different crystallographic orientation, as revealed by a different angle of extinction (Figure S1 from the Supporting Information). Based on these age relationships, Montero et al.^11^ proposed that the Cambrian–Ordovician granitic rocks crystallized from S-type magmas generated by anatexis of younger-than-600 Ma immature sediments sourced from Ediacaran igneous rocks.

REEs in rims and cores from SAB50 zircons have similar abundances, presenting more pronounced negative Eu anomalies than SAB51 zircons (Figure 3).

**Figure 5:**
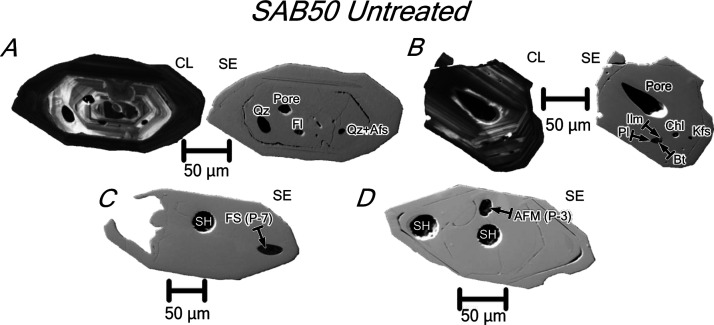
CL and SE images of untreated zircons from orthogneiss SAB50. The zircons show oscillatory zoning and an Ediacaran inherited core overgrown by a Cambro–Ordovician rim, with a core–rim interface outlined in many cases by a tiny cleft on the polished surface. Mineral inclusions in the zircons are restricted to the cores. (A) Zircon with oscillatory zoning hosting monomineralic inclusions of quartz and fluorite and a tear drop-shaped polymineralic inclusion of quartz + alkali feldspar. Note the variable orientation of the oscillatory zoning in the inner core. (B) Zircon with oscillatory zoning hosting a coarse pore, which may represent a gas bubble, a polymineralic inclusion of plagioclase + biotite + ilmenite, and monomineralic inclusions of chlorite and K-feldspar. Note that the oscillatory zoning of the core is not truncated by that of the rim. (C) Monomineralic inclusion of fluorosilicate with a composition close to that of fluorillite (see analysis in Table 2). (D) Monomineralic inclusion of a complex Mg–Fe–Ca–Na aluminofluoride mineral (probably hydrokenoralstonite; see analysis in Table 2; cf.,^9^). Mineral abbreviations after Whitney and Evans.^8^ Other abbreviations: AFM = aluminofluoride mineral; FS = fluorosilicate; and SH = SHRIMP analysis spot.

The Cambrian–Ordovician zircon rims are remarkably free of inclusions. In contrast, the Ediacaran cores often contain a plethora of mono- and polymineralic inclusions (Table 1). The former comprise quartz, K-feldspar, chlorite, apatite and fluorapatite, rutile, and hercynite. The latter consist of tear drop-shaped globules of quartz + alkali feldspar, quartz + K-feldspar + ilmenite, plagioclase + biotite + ilmenite, and quartz + monazite + fluorapatite (Figure 5A,B). Zircon cores also host up to 50 μm-long micropores that can represent gas bubbles (Figure 5B), suggesting the presence of a fluid during zircon growth. Also, we have found inclusions of F-rich minerals such as fluorite (Figure 5A), a fluorosilicate with a composition close to that of fluorillite (Figure 5C; Table 2) and a complex Mg–Fe–Ca–Na aluminofluoride mineral, probably hydrokenoralstonite (Figure 5D; Table 2).

### Syenite REG20

It is a silica-saturated syenite from the outer envelope of the 2.44 Ga Awsard kalsilite syenite intrusion of the west Reguibat Shield.^7^ The rock contains abundant euhedral to subhedral zircon grains that form short stubby prisms (80–130 μm long) terminated by short pyramids (Figure 1). REG20 zircons are translucent to opaque with different colors from yellow to purple and brown (Figure 1). They are highly metamict, especially in the cores, with a marked oscillatory zoning that is locally partially obliterated. They contain abundant inclusions and cracks that show a radial disposition in most cases (Figure 2C). In our experience, this appearance is typical in zircons that have suffered intense high-*T* hydrothermalism or fenitization. It is produced because zircon expands as it becomes metamict, and the amount of expansion depends on the intensity of metamictization. Therefore, less-metamict rims fracture if they surround a more expanded core. REG20 zircons are rich in U, Th, and Pb, both radiogenic and common. This is why we use them routinely in the SHRIMP IBERSIMS laboratory as a standard for mass calibration. In contrast with SAB51 and SAB50, REG20 zircons contain negligible W.

**Figure 6:**
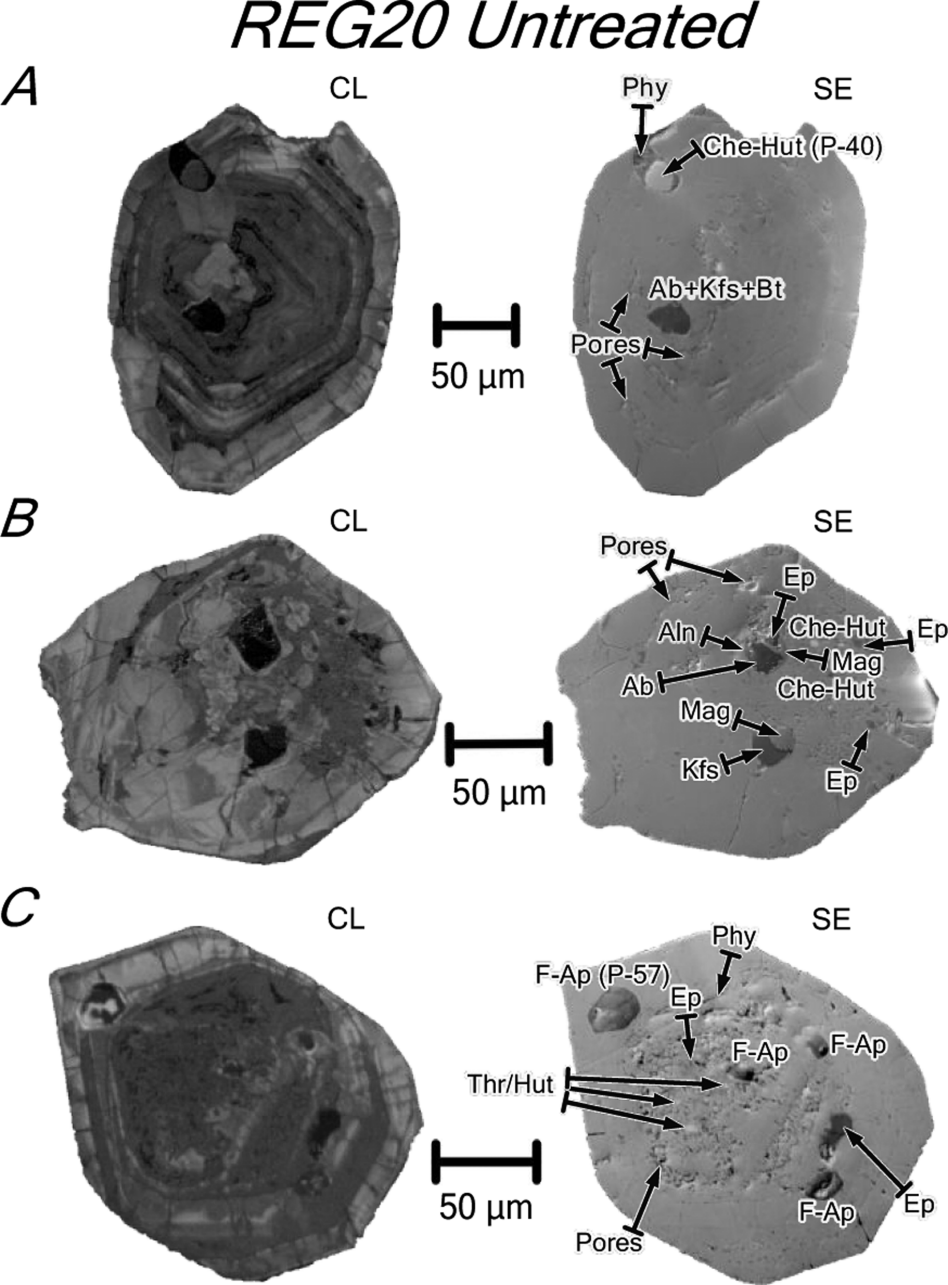
CL and SE images of untreated zircons from syenite REG20. The zircons are metamict, having oscillatory or patchy zoning with porous metamict zones in the core and in bands parallel to the zoning. (A) Zircon with oscillatory zoning and an inclusion of relatively coarse cheralite–huttonite solid solution (see analysis in Table 2) surrounded by a tiny aggregate of secondary phyllosilicates and polymineralic inclusions of biotite + albite + K-feldspar. (B) Zircon with patchy zoning and polymineralic inclusions of albite + magnetite + epidote + allanite and K-feldspar + magnetite and tiny inclusions of epidote, which appears to fill in microcracks, and cheralite–huttonite solid solution. (C) Zircon with oscillatory zoning and inclusions of F-bearing apatite (see analysis in Table 2), thorite/huttonite, and secondary phyllosilicates and epidote. Note the presence in the three grains of non-luminescent radial microcracks crosscutting the oscillatory zoning. Mineral abbreviations after Whitney and Evans.^8^ Other abbreviations: Che–Hut = cheralite–huttonite solid solution; Phy = phyllosilicates; and Thr/Hut = thorite/huttonite.

REG20 zircons have abundant inclusions (Table 1). These are euhedral to subhedral single crystals of fluorapatite, thorite/huttonite, and cheralite–huttonite solid solution or diverse associations of biotite + albite + K-feldspar, albite + magnetite + epidote + allanite, and K-feldspar + magnetite (Figure 6). Secondary phyllosilicates and epidote appear either as tiny grains or, more frequently, as veins infilling microcracks (Figure 6).

## 4 Results

### 4.1 Experimental Products

The mineral and glass inclusions produced in zircon grains during the heat treatment and their main textural features are summarized in Table 5. The major-element compositions of selected mineral phases and glasses are listed in Tables 2 and 6, respectively, and the trace-element compositions of the host zircons are reported in Tables 3 and 4.

**Table 5:** Summary of Mineral Assemblages and Textures in Annealed Zircon Grains^a^

run conditions: 1300 °C and 6 months				
run	starting material	grain number	mineral and glass inclusions	main textural features
E1	SAB51	268	LKRG (9)	massive or filling aligned micropore patches
			FSG^1^ (4)	elongated tear drop-shaped glass inclusions locally accompanied by gas bubbles or by euhedral baddeleyite + fluorite
			KRG (8)	
			Bdy (30)	euhedral shapes accompanied glass inclusions
			Bdy + Fl (2)	
			Sch and CMT (24)	anhedral shapes
E2	SAB50	260	LKRG (10)	(1) preferentially placed at the core–rim interface as continuous elongated zones or as micropore alignments; (2) replacing dark cathodoluminescent zones of the cores or as isolated tear drop-shaped bubbles, some of them partially filled with euhedral baddeleyite, fluorite, or both
			FSG^1^ (2)	
			FSG^2^ (5)	
			KRG (9)	
			CSG (3)	
			Bdy (31)	euhedral shapes accompanying glass inclusions
			Bdy + Fl (1)	
			F-Ap (13)	anhedral shapes
			Mnz (3)	
			Fl (2)	
			Sch and CMT (26)	
			Sri (1)	
E3	REG20	92	CSG (27)	anhedral shapes with abundant gas microbubbles
			Bdy (20)	anhedral shapes

a Mineral abbreviations after Whitney and Evans^8^. Other abbreviations: CSG = Calcic silicate glass; FSG^1^ = high-silica fluorosilicate glass; FSG^2^ = low-silica fluorosilicate glass; KRG = potassic rhyolite glass; LKRG = low-potassium rhyolite glass; CMT = Ca–Mg tungstate; and Sri = srilankite. The number of grains with the reported inclusions indicated in parentheses.

**Table 6:** Selected EDX Analyses of Glass Inclusions (wt %)

sample	SAB51, tonalite										REG20, syenite				
grain	1		2	3		4	5	6	7	8	1	2	3		
type^a^	FSG^1^	KRG	KRG	KRG		FSG^1^	LKRG	LKRG	LKRG	LKRG	CSG	CSG	CSG		
point	435	440	448	456	453	151	120	55	148	49	179	293	184	187	186
SiO_2_	76.8	77.0	73.9	76.0	76.4	75.0	79.1	83.1	80.6	87.1	65.5	54.1	64.6	66.9	67.8
TiO_2_				0.23							0.38		0.78	0.78	0.72
Al_2_O_3_	14.2	14.0	16.3	14.9	14.8	13.9	16.7	13.6	15.5	10.8	7.05	5.93	7.03	6.30	6.82
FeO															
MgO							0.69	0.41	0.57		2.83	2.25	2.40	2.05	2.34
CaO	2.96	2.88	2.20	2.05	2.09	1.57	1.91	1.41	1.70	0.94	21.3	23.3	20.4	17.6	19.2
Na_2_O	1.94	1.89	1.56	1.86	1.86	2.25	1.41	1.29	1.47	0.95				0.14	
K_2_O	4.12	4.24	5.44	4.95	4.91	4.37	0.18	0.19	0.19	0.16					
P_2_O_5_												1.42			
Y_2_O_3_														2.58	
Ce_2_O_3_												2.32	0.58		
ThO_2_											2.98	10.6	4.28	3.68	3.16
F						5.02									
Cl			0.62												
total	100	100	100	100	100	102	100	100	100	100	100	100	100	100	100
–O≡F						2.12									

sample	SAB50, orthogneiss														
grain	1	2	3	4	5	6	7		8		9	10	11		12
type^a^	FSG^1^	FSG^2^	FSG^2^	KRG	KRG	KRG	LKRG		KRG	CSG	LKRG	LKRG	LKRG		CSG
point	15	127	131	185	191	275	212	215	166	167	5	189	195	193	175
SiO_2_	76.7	33.4	24.1	77.1	78.1	76.8	82.5	83.2	80.7	57.4	84.4	74.5	81.6	82.0	63.7
TiO_2_		1.06			0.33							0.28	0.48		
Al_2_O_3_	15.4	14.6		13.2	13.5	14.2	14.5	14.7	11.6	24.4	12.3	15.8	13.0	13.2	24.7
FeO				1.24		0.38		0.36							
MgO	0.54	3.89	14.4			0.72					0.35	4.93			2.09
CaO	0.67	30.9	43.3	0.63	0.53	0.77	0.31	0.24	0.66	17.15	1.72	2.08	1.11	1.16	8.37
Na_2_O	1.36			2.48	1.69	2.45	1.48	0.28	0.99	1.08	0.95	0.73	1.90	1.73	1.23
K_2_O	3.46			5.32	5.79	4.67	1.24	1.18	6.07		0.23	0.25	1.91	1.92	
P_2_O_5_		1.44													
Y_2_O_3_		2.25													
Ce_2_O_3_		0.95													
Nd_2_O_3_		0.73													
F	1.69	18.7	31.7												
Cl	1.15														
total	101	108	113	100	100	100	100	100	100	100	100	100	100	100	100
–O=F	0.71	7.89	13.3												
–O=Cl	0.26														

a CSG: calcic silicate glass; FSG^1^ = high-silica fluorosilicate glass; FSG^2^ = low-silica fluorosilicate glass; KRG = potassic rhyolite glass; and LKRG = low-potassium rhyolite glass.

**Figure 7:**
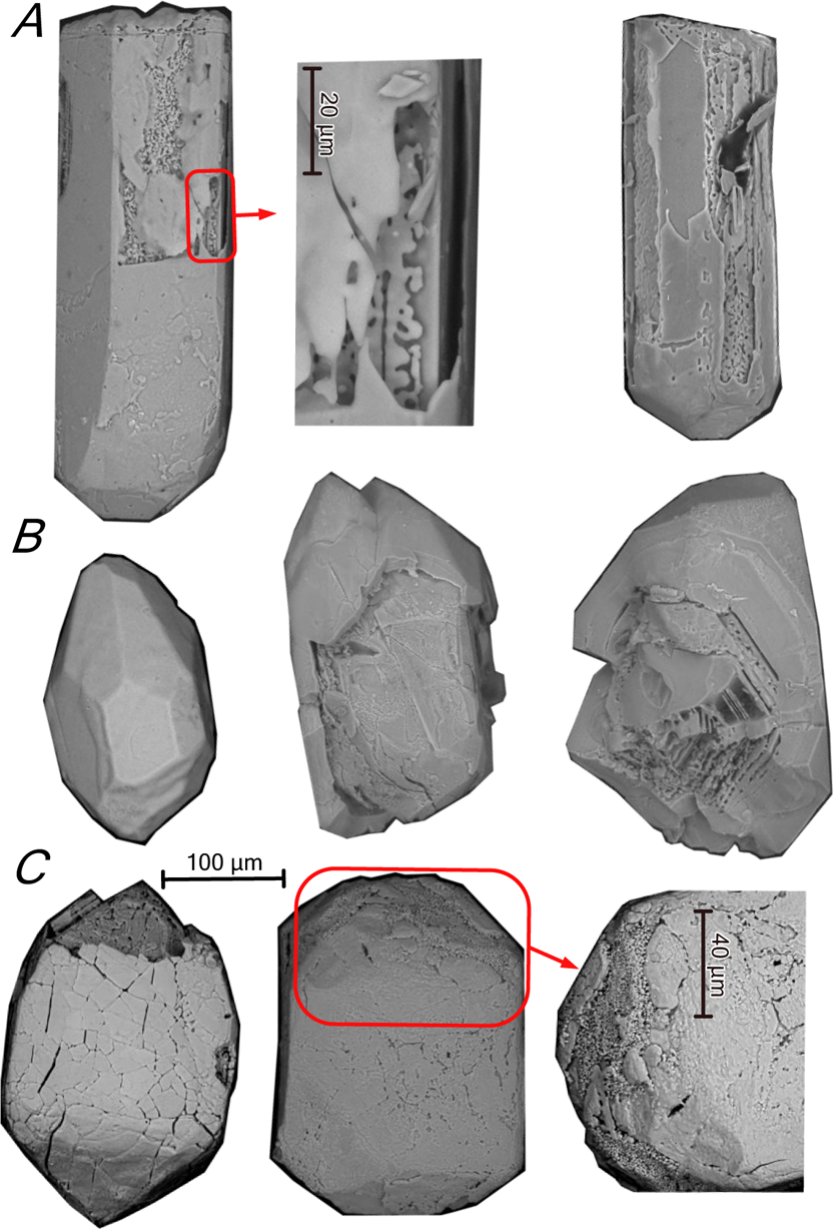
SE images of annealed zircons from tonalite SAB51 (A), orthogneiss SAB50 (B), and syenite REG20 (C). Annealed zircons have acquired an onion-like structure with alternating little altered and heavily recrystallized, even partially melted zones.

All annealed zircon grains show extensive melting and recrystallization in the darkest CL zones, especially if they are metamict. This causes some zircon grains to acquire an onion-like structure with alternating unaltered (or little altered) and heavily recrystallized, even partially melted zones (Figure 7).

**Figure 8:**
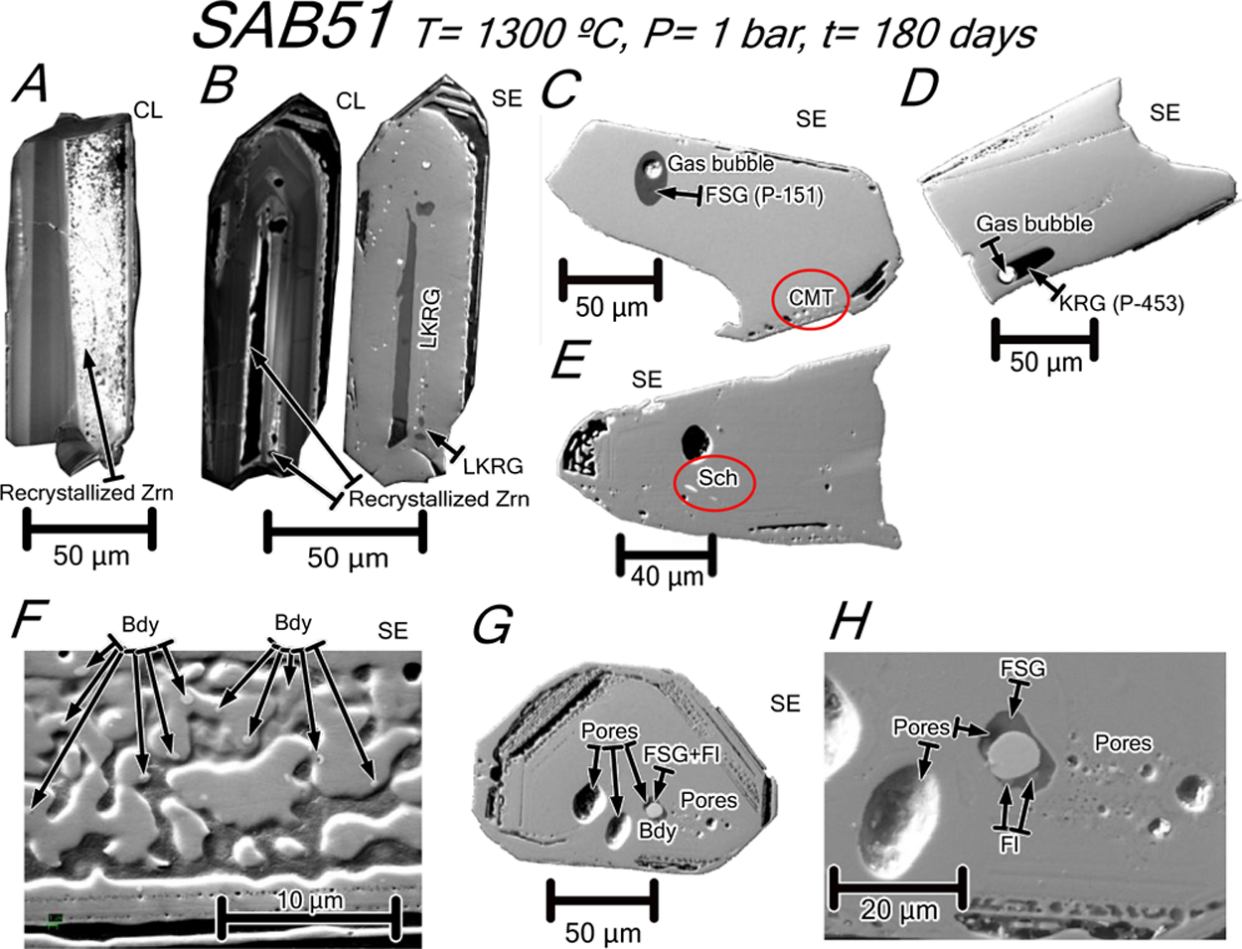
CL and SE images of annealed zircons from tonalite SAB51. (A,B) Zircons showing that the bands parallel to prismatic and pyramidal faces become luminescent after heat treatment suggesting recrystallization. Note in (B) a large inclusion of low-*K* rhyolitic glass filling the micropores of the bands. (C) Zircon hosting a tear drop-shaped fluorosilicate glass inclusion (see analysis in Table 6) with a gas bubble. Note the presence of very tiny inclusions of bright Ca–Mg tungstates (red oval). (D) Zircon hosting an elongated tear drop-shaped potassic rhyolite glass inclusion (see analysis in Table 6) with a gas bubble. (E) Zircon hosting a scheelite inclusion (red oval). (F) Zircon with very tiny baddeleyite grains included in zircon in contact with an empty space which possibly contained glass. (G) Zircon hosting micropores and a multi-phase inclusion of fluorosilicate glass + fluorite + euhedral baddeleyite with a micropore. (H) Detail of the multi-phase inclusion shown in G. Mineral abbreviations after Whitney and Evans.^8^ Other abbreviations: CMT = Ca–Mg tungstate; FSG = fluorosilicate glass; KRG = potassic rhyolite glass; and LKRG = low-*K* rhyolite glass.

The slightly metamict dark cathodoluminescent bands of SAB51 zircons become highly luminescent because of zircon recrystallization (Figures 8 and S2 from the Supporting Information), forming minute zircon grains and open spaces that either remain empty (Figures 8A and S2C) or are filled with low-*K* rhyolitic glass (Figures 8B,F and S2D–G). The glass patches can be massive, especially if there were polymineralic inclusions (Figure 8B,F), or form narrow and elongated veins (Figure S2E,G). Zircon surrounding the glass has blob-like shapes that suggest contact between two highly viscous fluids (Figures 8F and S2D–G), melt and plastic viscous zircon, and proves that the glass was derived from the melt that was generated during the experiments. The zircon blobs may include very tiny (less than 1 μm long) baddeleyite grains (Figure 8F). Besides, there are 20–30 μm rounded to elongated tear drop-shaped inclusions of fluorosilicate glass and potassic rhyolite glass, locally accompanied by gas bubbles, and of fluorosilicate glass + euhedral baddeleyite + fluorite (Figure 8C,D,G,H).

**Figure 9:**
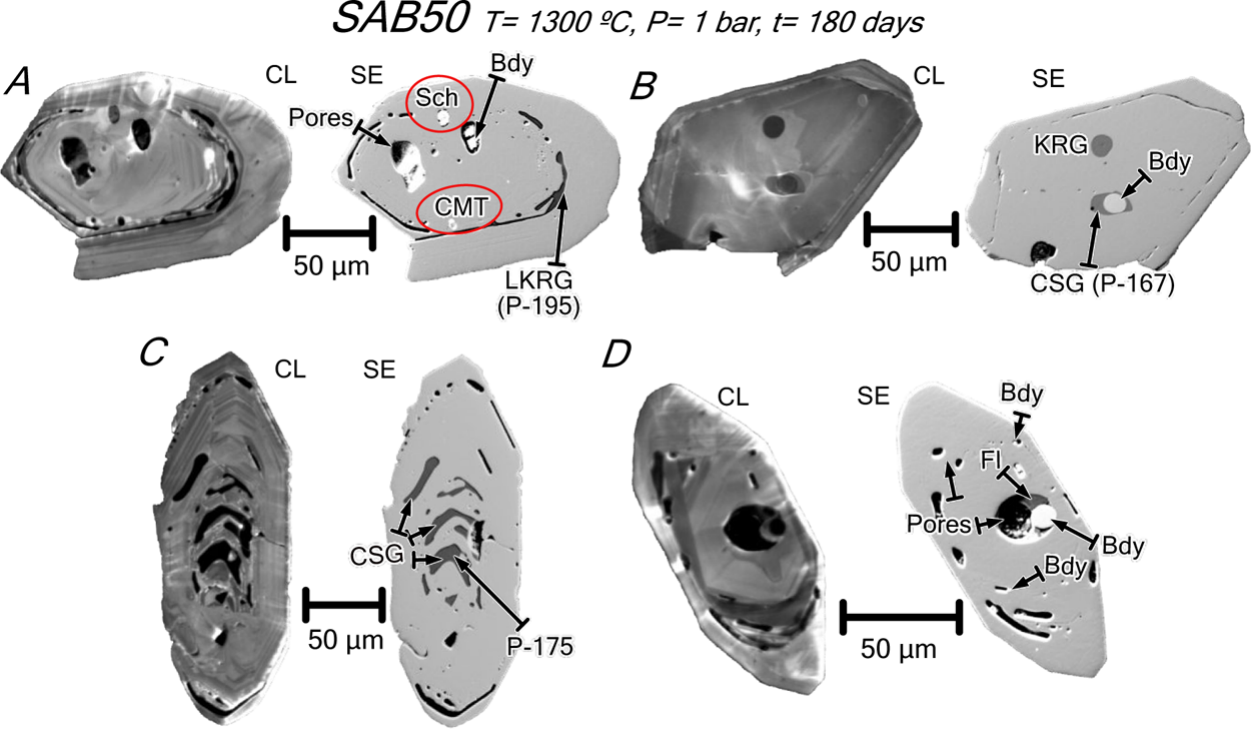
CL and SE images of annealed zircon grains from orthogneiss SAB50. (A) Zircon hosting monomineralic inclusions of Ca–Mg tungstates, scheelite (red ovals), and baddeleyite and low-*K* rhyolite glass inclusions lying along the core–rim interface. (B) Zircon with oscillatory zoning and a shell rimming a core that hosts a tear drop-shaped potassic rhyolite glass inclusion and a multi-phase inclusion of euhedral baddeleyite + calcic silicate glass (see analysis in Table 6). (C) Zircon with abundant inclusions of calcic silicate glass (see analysis in Table 6) parallel to the compositional zoning of the core. (D) Zircon with oscillatory zoning and a shell rimming a core that hosts a tear drop-shaped multi-phase inclusion of fluorite + euhedral baddeleyite with a micropore. Note also the occurrence of baddeleyite inclusions in elongated micropores with rounded tips opened mostly along the core–rim interface. Mineral abbreviations after Whitney and Evans.^8^ Other abbreviations: CSG = calcic silicate glass; FSG = fluorosilicate glass; KRG = potassic rhyolite glass; LKRG = low-*K* rhyolite glass; SH = SHRIMP analysis spot; and CMT = Ca–Mg tungstate.

The annealed zircon grains of the SAB50 orthogneiss host inclusions of glass, baddeleyite, fluorapatite, monazite, fluorite, scheelite, and a few grains of srilankite, of which the last two were never found in the untreated grains (Figures 9 and S3 from the Supporting Information; Table 5). Glass inclusions are the most abundant; they appear preferentially placed at the core–rim interface—either as micropore alignments or, much more frequently, as continuous and relatively voluminous elongated zones that indicate considerable melt migration to these core–rim interfaces (Figures 9A and S3A). Glass inclusions also appear replacing dark cathodoluminescent zones of the cores or as isolated 10–20 μm tear drop-shaped globules. Some of them are partially filled with euhedral baddeleyite, fluorite, or both (Figures 9A,B,D and S3B). The compositions of the glasses (see the next section) range from low-*K* rhyolitic, mostly placed at the core–rim interfaces, to high-K or Ca-rich silicic glasses in the tear drop-shaped globules.

Another notable fact of annealed zircons from both SAB50 and SAB51 is the conspicuous presence of inclusions of Ca–Fe–Mg tungstates (Figures 8C,E, 9A, and S3C) that we never found in untreated zircons despite an extensive search with SEM.

**Figure 10:**
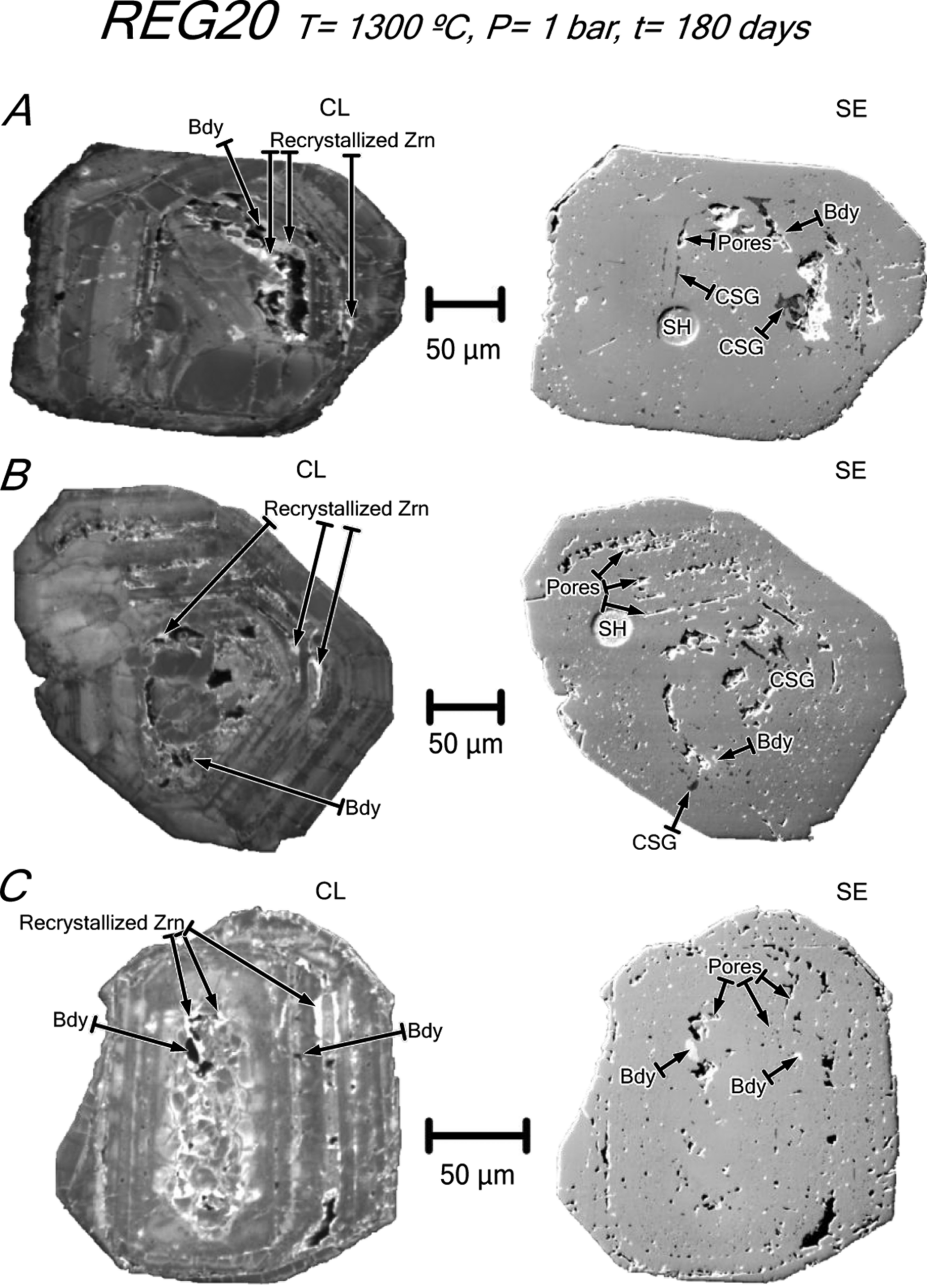
CL and SE images of annealed zircon grains from syenite REG20. (A–C) Zircons showing that the porous metamict domains become luminescent due to recrystallization and are filled by calcic silicate glass with abundant gas microbubbles. Note also the occurrence of anhedral baddeleyite in recrystallized domains and the absence of Th-REE accessory phases that were most probably dissolved into the calcic silicate glass (see text for discussion); microcracks also turn luminescent after heat treatment. Mineral abbreviations after Whitney and Evans.^8^ Other abbreviations: CSG = calcic silicate glass and SH = SHRIMP analysis spot.

The annealed zircon grains of the REG20 syenite changed markedly compared to the unheated grains. Most of them were fractured during the recovery because of the enlargement of their previous fractures. The porous metamict zones from the grain core, the bands parallel to oscillatory zoning, and the microcracks became luminescent. Inclusions mostly consist of glass and relatively coarse (up to 10 μm long) anhedral baddeleyite (Figure 10A–C; Table 5). The glass is calcic and silica-rich with abundant gas microbubbles. Remarkably, the Th-REE accessory phases, which are common in the untreated zircon, disappear after annealing. This implies that they must have been dissolved into the calcic silicate glass, as suggested by the significant Th concentration in the glasses (Table 6). As described in Section 4.4, one of the most surprising effects observed in the REG20-annealed zircon was the disappearance of the common lead.

### 4.2 Composition of Glass Inclusions

**Figure 11:**
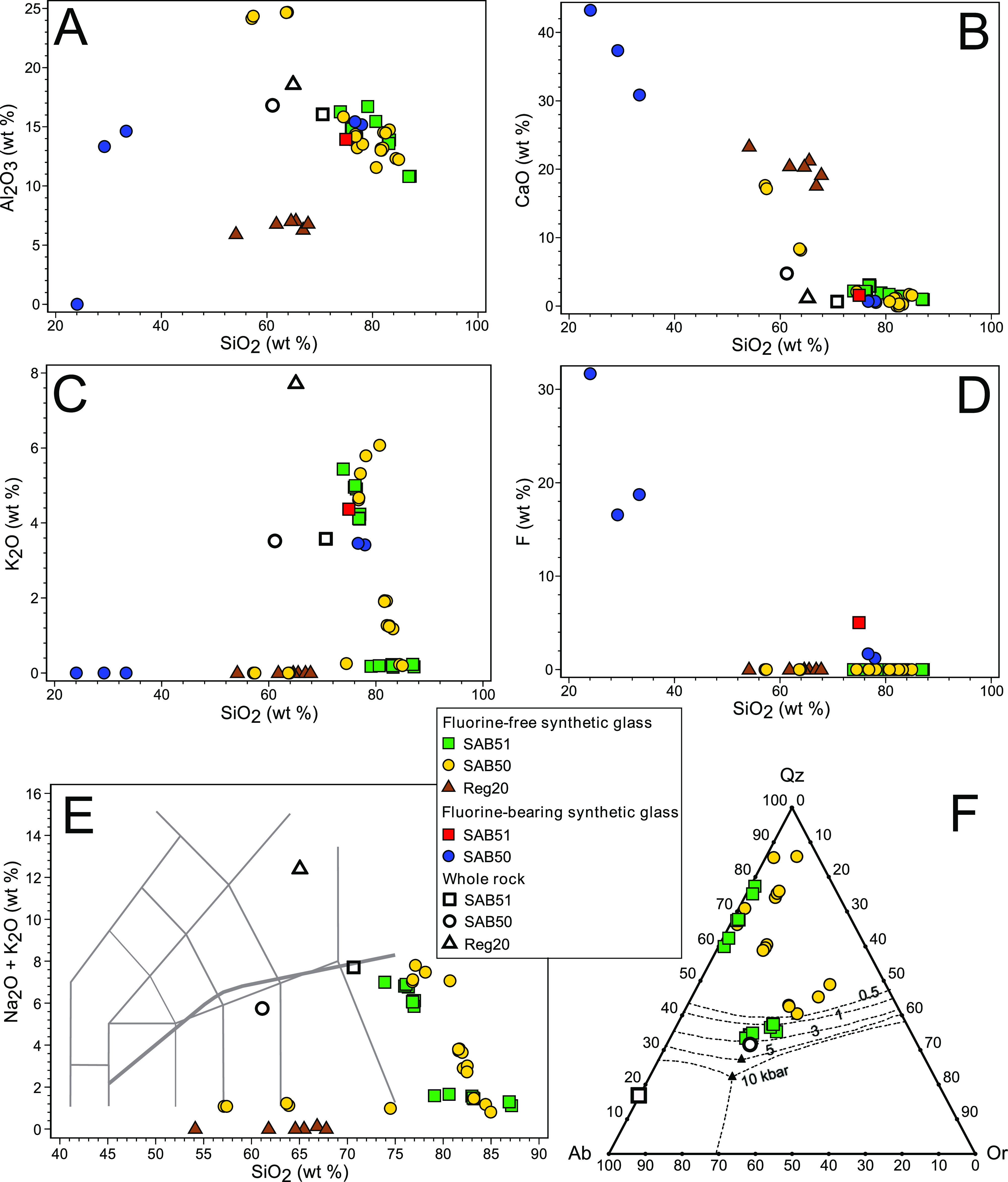
Composition of glass inclusions of the annealed zircon grains from tonalite SAB51, orthogneiss SAB50, and syenite REG20. (A) Al_2_O_3_ vs silica. (B) CaO vs silica. (C) K_2_O vs silica. (D) Fluorine vs silica. (E) Total alkalis vs silica (TAS) diagram. (F) Phase equilibrium diagram of the Qz-Ab-Or ternary projected from anorthite following the procedure of Blundy and Cashman;^12^ cotectic lines drawn from experimental data by Tuttle and Bowen,^13^ Luth et al.,^14^ and Ebadi and Johannes.^15^

Four compositional types of glasses can be identified in the annealed zircon grains (Figure 11; Table 6): (i) fluorosilicate, (ii) low-*K* rhyolite, (iii) potassic rhyolite, and (iv) calcic andesite to dacite. The first three types appear in samples SAB50 and SAB51. The last kind of glass occurs in samples SAB50 and REG20.

There are two types of *fluorosilicate glasses*, one with a high-silica rhyolite composition (silica range: 75–78 wt %) having 14–15 wt % Al_2_O_3_, <1.6 wt % CaO, 1.2–2.2 wt % Na_2_O, 3.4–4.4 wt % K_2_O, and fluorine ranging from 1.2–1.7 wt % in sample SAB50 to ca. 5 wt % in sample SAB51 and the other, which only appears in sample SAB50, with low silica (24–33 wt %), high CaO (31–43 wt %) and fluorine (17–32 wt %), and variable Al_2_O_3_ (0–15 wt %) and MgO (3.6–14 wt %).

The *potassic rhyolite* glasses (>4.1 wt % K_2_O) show a composition close to that of the fluorine-bearing high-silica rhyolites, with 75–78 wt % SiO_2_, 12–16 wt % Al_2_O_3_, 0.53–3.1 wt % CaO, and 0.99–2.5 wt % Na_2_O.

The *low-K rhyolite glasses* (<1.9 wt % K_2_O) have more variable silica (range: 75–87 wt %), similar alumina (range: 11–16 wt %), <2.1 wt % CaO, and 0.28–1.9 wt % Na_2_O.

The *calcic andesite* to *dacite glasses* (up to 23 wt % CaO) contain 54–68 wt % SiO_2_, variable alumina (range: 6–25 wt %), low Na_2_O (<1.2 wt %), and negligible K_2_O. The glasses included in REG20 zircons are variably enriched in ThO2 (range: 3.0–11 wt %) and contain up to 2.3 wt % Ce_2_O_3_, 1.2 wt % Nd_2_O_3_, and 3.5 wt % Y_2_O_3_.

The compositions of *potassic rhyolite* glasses included in SAB50 and SAB51 zircons are slightly more enriched in silica and K_2_O and depleted in alumina than those of the corresponding host rocks (Figure 11). This is consistent with the expectation that the glass inclusions represent evolved residual melts entrapped in zircon during its growth. However, the composition of the other glass types can hardly be related to the host-rock composition by simple magma fractionation processes, and therefore, they represent the products of either mineral inclusion melting or the interaction of zircon with the silica sealant, as discussed below.

### 4.3 Composition of Recrystallized Zircons

The Ti and other trace-element concentrations of the SAB51 and SAB50 zircons can be easily determined using LA-ICP-MS because they have large areas free of inclusions. Unfortunately, this is not the case for REG20 zircons, in which we could not obtain a single clean LA-ICP-MS measurement.

The comparison between untreated and annealed SAB51 and SAB50 zircons reveals that the annealed ones show perceptible impoverishments in LREE except for Ce, which remains constant or increases slightly. This effect causes enhanced Ce anomalies in the two samples (Figure 3). However, in some zircon grains, both annealed and untreated, these anomalies are less intense or even absent, due to a higher La content, which may be caused by alteration processes.

**Figure 12:**
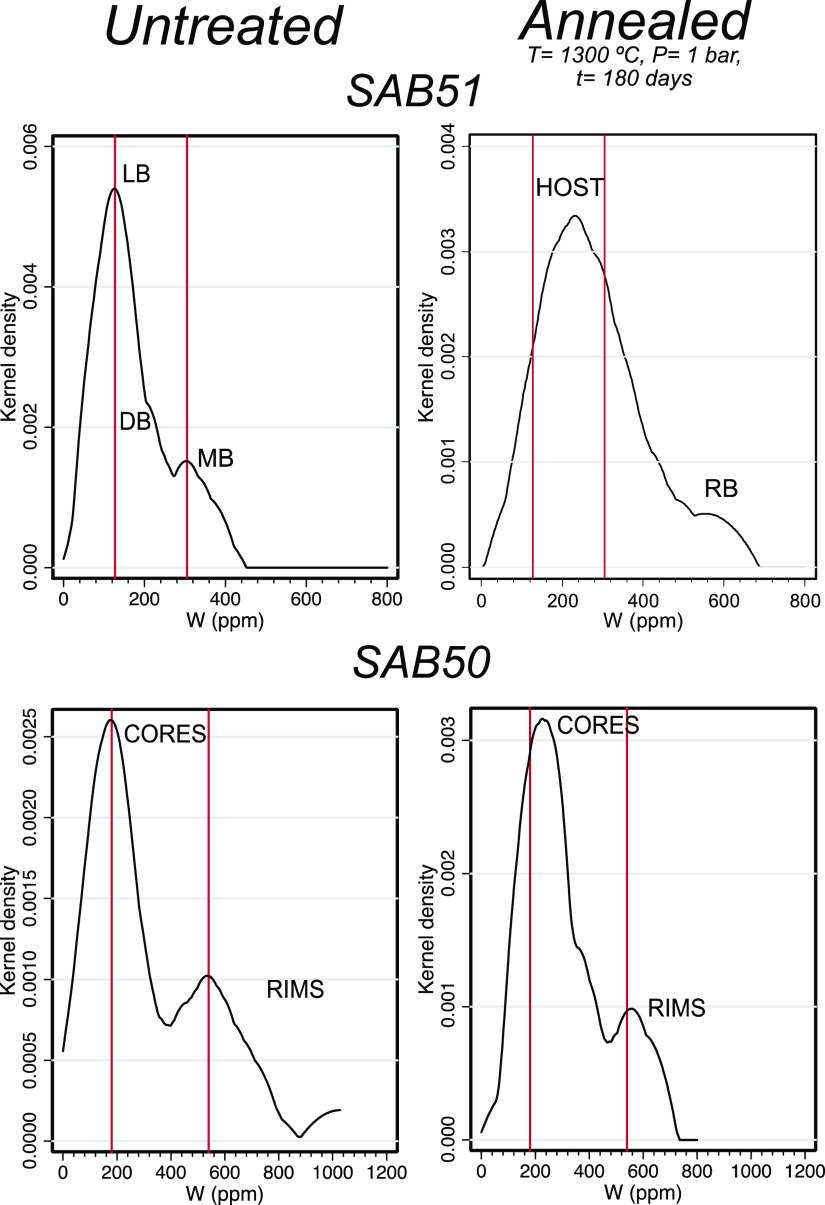
Tungsten distribution of untreated and annealed zircon grains in the tonalite SAB51 and orthogneiss SAB50. Note that the untreated SAB51 zircons show two peaks, for light and dark bands (104 and 164 ppm, respectively) and a peak for metamict bands (305 ppm), whereas annealed ones show no difference between light and dark bands, thus indicating homogenization, but there are still differences between host and recrystallized bands. Untreated SAB50 zircons have a clear difference in W between cores and rims (177 and 570 ppm, respectively), remaining practically unchanged in the annealed ones (cores: 225 ppm, rims: 555 ppm). Abbreviations: LB = light bands, DB = dark bands, MB = metamict bands, and RB = recrystallized bands.

In annealed SAB51 zircons, the light and dark bands show little differences in W concentrations, with average values of, respectively, ca. 267 ppm (range: 193–347 ppm) and ca. 217 ppm (range: 138–392 ppm), whereas recrystallized domains are W-richer (av. 440 ppm; range: 326–554 ppm; Figure 12, Table 4). In SAB50 zircons, cores and rims from annealed grains show more similar W concentrations (core: av. 219 ppm, range: 124–347 ppm; rim: av. 424 ppm, range: 200–601 ppm) than untreated ones (Figure 12, Table 4).

**Figure 13:**
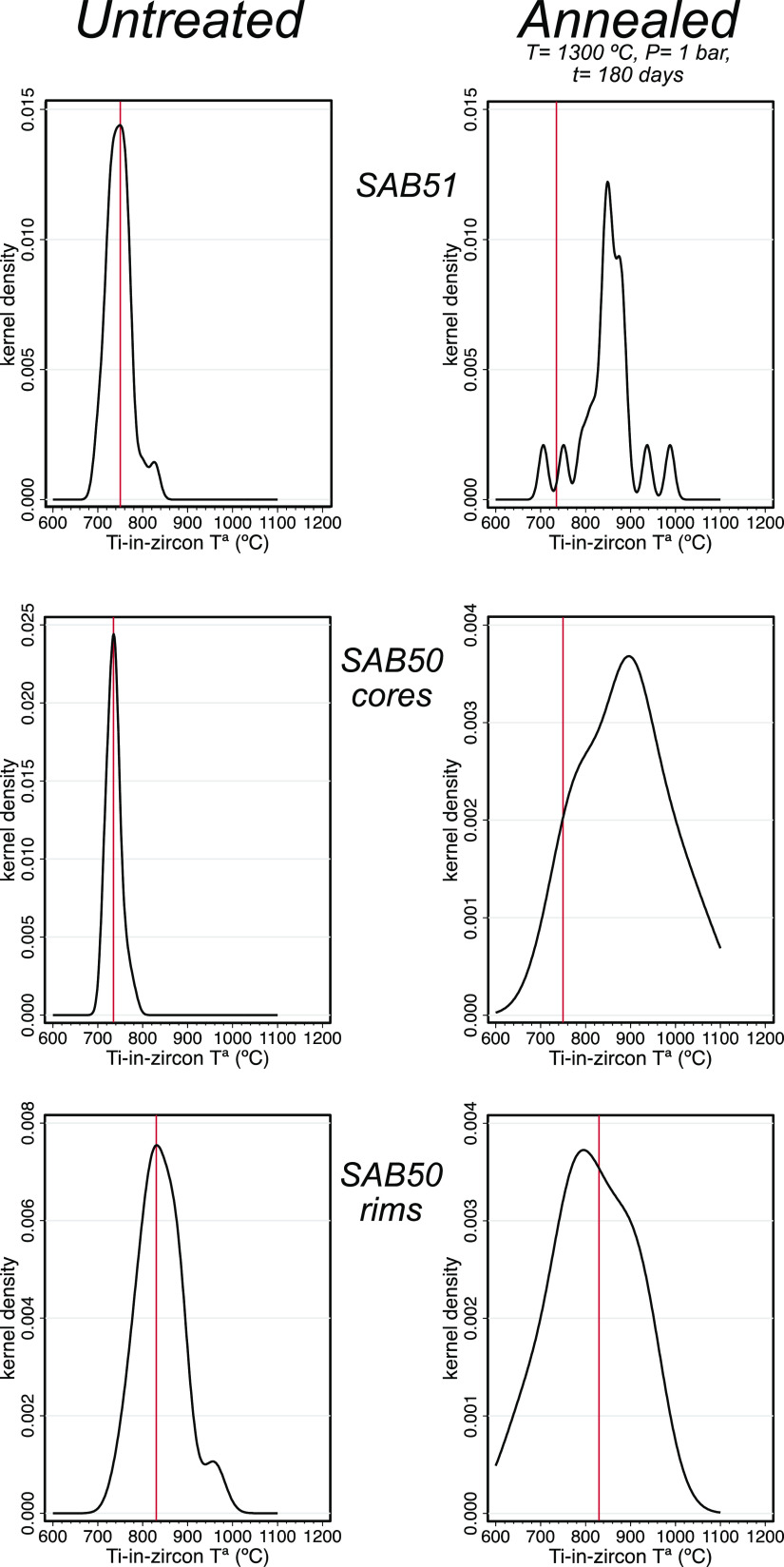
Distribution of Ti-in-zircon temperatures calculated with the expression of Watson and Harrison^17^ for untreated and annealed zircon grains from samples SAB50 and SAB51. Note that untreated SAB51 and Ediacaran cores of untreated SAB50 peak similar (about 750 and 740 °C, respectively), whereas Cambrian–Ordovician rims of untreated SAB50 peak at 830 °C. When annealed, the Ti-in-zircon temperatures for SAB51 and SAB50 cores increase to peak values around 800–900 °C, but Ti-in-zircon temperatures for SAB50 rims decrease slightly, peaking at 800 °C.

In the annealed zircons, the concentration of Ti increases slightly and the distribution of Ti-in-zircon temperatures^10,16^ widens in all cases; the SAB51 and SAB50 cores increase to peak values around 800–900 °C, but the SAB50 rims decrease slightly, peaking at ca. 800 °C, that is, lower than the cores, thus reversing the situation observed in the untreated crystals (Figure 13).

### 4.4 Common Pb Leaching

SAB50 and SAB51 zircons contain negligible common Pb, and their U–Pb SHRIMP results are, in general, concordant, with little or no radiogenic Pb loss. Heating experiments performed by Bea et al.^2^ revealed that zircons that are not in contact with the melt do not lose radiogenic Pb but homogenize their concentration within the zircon, disturbing the ^206^Pb/^238^U and ^207^Pb/^235^U ratios, and causing discordances only if the zircon was chemically heterogeneous.

However, the situation is remarkably different for REG20 zircons. In these, the intense metamictization, the elevated abundance of common Pb, and loss of radiogenic Pb fully prevent U–Pb SHRIMP dating. The geological position of the REG20 syenite in the outer rim of the funnel-shaped Awsard intrusion suggests that its age should be close to that of the nepheline and kalsilite syenites of the body’s interior, the zircons from which yielded SHRIMP U/Pb ages of 2459 ± 11 Ma (MSWD = 2.9) and 2458 ± 8 Ma (MSWD = 1.2), respectively.^7,18^

**Figure 14:**
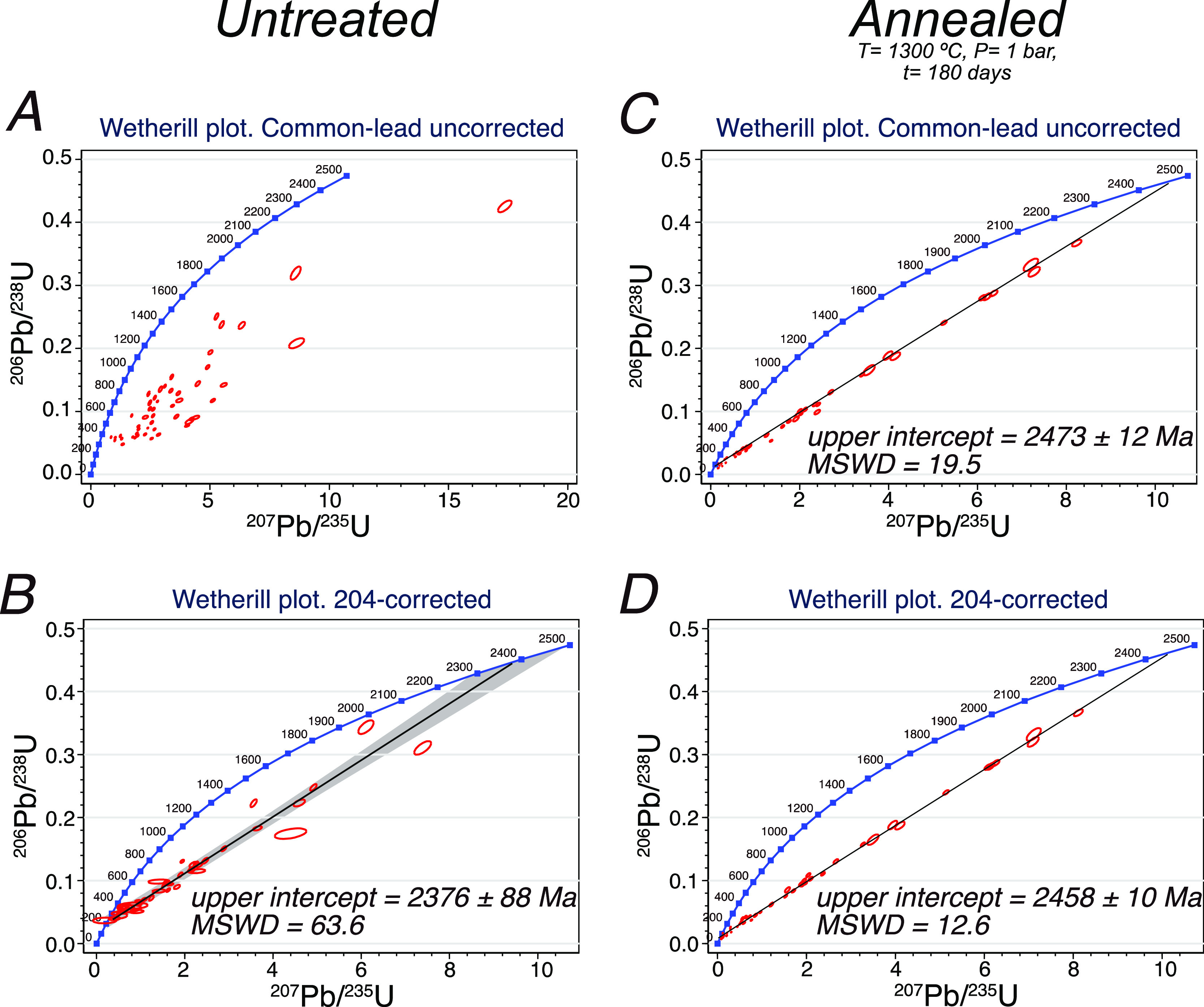
Wetherill plot of SHRIMP data obtained from untreated and annealed zircon grains of syenite REG20. (A) Wetherill plot with common lead uncorrected for untreated REG20 zircons. Note that the points define a cloud from which no sensible age can obtained. (B) Wetherill plot with the 204-correction for untreated REG20 zircons. Note that there is an upper intercept of 204-corrected ages at 2376 ± 88 Ma. (C) Wetherill plot with common lead uncorrected for annealed REG20 zircons. Note that the annealed zircons yielded a well-fitted common lead uncorrected discordia with an upper intercept at 2473 ± 12 Ma. (D) Wetherill plot with the 204-correction for annealed REG20 zircon. Note that the 204-corrected ages improve to 2458 ± 10 Ma (see text for discussion). Data reported in Tables S2 and S3 from the Supporting Information.

Figure 14A shows the SHRIMP U–Pb data, common Pb-uncorrected, that we got for this sample. The points define a cloud from which no sensible age can be obtained. Using the Cumming and Richards (1975)^19^ common Pb evolution scheme, we got an upper intercept of the 204-corrected ages at 2376 ± 88 Ma (MSWD = 63.6) (Figure 14B), which is unacceptable as a crystallization age. In contrast and very surprisingly, the annealed zircons yielded a well-fitted common lead-uncorrected discordia with the upper intercept at 2473 ± 12 Ma (MSWD = 19.5) (Figure 14C). The 204-correction improves the result up to 2458 ± 10 Ma (MSWD = 12.6) (Figure 14D), which is identical to the kalsilite and nepheline syenite zircons mentioned above, and is within the errors of the uncorrected discordia. Additionally, the lower intercept decreases from ca. 250 Ma in the untreated, to about 60–90 Ma in the annealed zircons. This indicates that some radiogenic lead was likely also lost.

According to numerical models by Bea and Montero,^1^ the massive common Pb loss leading to well-fitted discordias occurs at *T* > 1100 °C but not below, even when the zircons suffer extensive recrystallization. This suggests that common Pb dissolved into the melt migrating through the crystal. If the melt is not formed, the common Pb remains similar to the unheated zircons in spite of the annealing of the zircon.

## 5 Discussion

### 5.1 Inferences on the Crystallization of Zircon from the Variscan, Cambrian–Ordovician, and Ediacaran Magmatism

The analysis of glass inclusions in zircon is a valuable method for determining zircon-saturated melt compositions (^20^). In the untreated SAB50 and SAB51 zircons, the tear drop shapes of quartz + K-feldspar-bearing inclusions suggest that they were originally granite-melt inclusions. During heating, they gave rise to the potassic rhyolite glass (Table 6) with compositions lying along the quartz-feldspar cotectic valley of the Qz-Ab-Or ternary (Figure 11F). This fact suggests that zircon grains from the two samples grew from granite residua at low pressure, which is consistent with their geological mode of occurrence.

Additionally, the occurrence of gas bubbles plus fluoride and silicate minerals hosted in the untreated zircon grains from the two samples suggests the presence of a fluorine-rich fluid phase during zircon growth. This fact implies a high fluorine activity in the late magmatic fluids occurring during Ediacaran and Variscan times.

Elevated W concentrations in zircon, similar to SAB50 and SAB51 zircons, have never been reported in the literature to the best of our knowledge. This peculiarity of SAB50 and SAB51 zircons is likely related to the tungsten deposits that are abundant in Western Spain, particularly in the Sanabria zone, where we collected the samples SAB50 and SAB51.^21,22^

### 5.2 Contrasting Behavior of Ti and W in Zircon with Increasing Temperature

Under the redox conditions prevailing in Earth’s crust, Ti mainly occurs as a tetravalent ion. Ti^4+^ (ionic radius: 0.42 Å) may be incorporated into the zircon lattice either as ^IV^Ti^4+^ within silica tetrahedra, substituting for ^IV^Si^4+^ (ionic radius: 0.26 Å), which is the dominant mechanism,^16^ or as ^VIII^Ti^4+^ (ionic radius: 0.74 Å) within the ZrO_8_ triangular dodecahedra, replacing ^VIII^Zr^4+^ (ionic radius: 0.84 Å). In both cases, the substitution is homovalent, so that no compensating ion is required; however, the large differences in ionic radii make the substitution difficult. This is why Ti concentration in zircon is always low and increases with the temperature, yielding a useful thermometer.^16,17^ The increased Ti concentrations of the annealed SAB50 Ediacaran cores and SAB51 zircons over their untreated equivalents suggest that some Ti has been incorporated into the zircon lattice during heating. We propose that the Ti sources were minute Ti-rich inclusions, mainly rutile, which abound.

Under the redox conditions prevailing in Earth’s crust, W mainly occurs as a hexavalent ion, W^6+^ (ionic radius: 0.42 Å in fourfold coordination); it can only replace ^IV^Si^4+^ within the zircon lattice. Therefore, the incorporation of W^6+^ requires the coupled incorporation of a divalent cation, likely Ca^2+^, Mg^2+^, or Fe^2+^, to compensate charges. The compensating ion necessarily replaces ^VIII^Zr^4+^ within the ZrO_8_ triangular dodecahedra. However, this substitution disturbs the zircon structure because of the large differences of ionic charge and radius between ^VIII^Zr^4+^ and any potential compensating ion.

Upon annealing, the overall abundance of W also increases relative to the untreated equivalents, as evidenced by concentration values above the detection limit in all W analyses of the treated zircons (Figure 12; Table 4). These relationships could be explained in an analogous way to Ti, that is, by the dissolution of minute W-mineral impurities, mostly concentrated in the CL darkest and more metamict areas. However, because of the more limited solubility of W in the zircon lattice (mostly in the order of hundred ppm), the zircon rejects part of W and the coupled divalent cations (Ca, Fe, and Mg) to form the new tungstate inclusions, mostly scheelite, seen in the annealed SAB50 and SAB51 zircons.

Tungsten analytical chemistry in silicate rocks at the low-ppm level required for ore deposit prospecting is complex because of the lack of sensitivity of most techniques, the isobaric interferences by Er oxides in ICP–MS, and the W contamination produced by most grinding systems. In contrast, identifying minute W concentration in zircon with an electron microprobe or scheelite inclusions in SEM with EDAX is fast and straightforward. Therefore, we suggest that either direct W analyses of zircon in thin sections or annealing zircons and looking at them for scheelite and other tungstate inclusions offer considerable potential as tools for searching for W deposits.

### 5.3 Permeability Creation by Zircon Annealing: Evidence for an Open-System Behavior

Natural zircon can be permeable because of transport pathways related to a network of microcracks and micropores present in metamict domains (e.g., ref (23)). Recrystallization of metamict zircon by annealing, which occurs quickly at *T* > 900 °C (e.g., refs (2) and (24)), increases permeability in zircon because of the formation of nanopores caused by significant volume reduction of the recovered domains.^23,25,26^ This micropore network can explain the migration and accumulation of low-*K* rhyolite melts in the porous bands parallel to either the pyramidal or prismatic faces of SAB51 zircons.

An interesting case is the accumulation of melt at the core–rim boundaries of the composite zircon crystals of sample SAB50, which was previously noted by Bea et al.^2^ (Figure 9). Zaraisky and Balashov^27^ and references therein realized that the volume increase resulting from thermal expansion of a given rock is larger than the sum of the volume increase of its constituent minerals because of the creation of additional intergranular pore space rocks upon heating. The reason is that thermal expansion is a tensorial property, and the rock minerals are variously orientated. The same effect seems to occur inside a composite zircon grain in which the core and rim have different crystallographic orientations. The resulting anisotropic thermal expansion opens up pore spaces at the core–rim interface that therefore may act as an attractor for the mobile melt, being concomitantly filled by molten inclusions, whose migration through the crystal can take place either along micropores and microcracks or through the crystal lattice itself in the way described by Schiano et al.^28^

### 5.4 Leaching of Impurities by the Migrating Melt

Trace-element and mineral impurities in zircon are primarily concentrated in metamict domains, and these are prone to melt and develop high porosity during annealing. This is the case for REG20 zircons (Figure 10), which formed abundant microveins of calcic andesite to dacite melts. The melt migration through the recrystallizing crystal permitted the effective dissolution of U, Th, and REE accessory phases, which disappear from the annealed crystals and cause thereby high abundance of these chemical components in the calcic andesite to dacite glasses that fill the micropores and cracks.

Of special interest is the leaching of common Pb (see Figure 14) because of the favorable influence of this process on the dating of zircon. Several authors have shown that Pb in old and metamict zircons tends to form nanospheres of metallic Pb or Pb oxides.^29−32^ Metallic Pb and oxides are highly soluble in silicate melts, where Pb^2+^ behaves like a network modifier if its concentration is below the level of a few percent.^33^ Given that the recrystallized zircon domains lost almost all of their common Pb, we suggest that it mostly dwelt in the nanospheres or other phases that were easily soluble in the melt. In contrast, radiogenic Pb is mainly retained in the zircon lattice; only minor amounts move to the melt as indicated by the lower intercept age of discordia lines from annealed zircons compared to those from untreated zircons. This observation provides a way for dating highly metamict zircons, which is otherwise impossible because of the combination of radiogenic Pb loss and common Pb gain.

The observation also fits well with the work of Mattinson,^34^ who showed that combining annealing and multi-step HF leaching of zircon concentrates markedly decreases the discordance caused by common lead and lead loss in TIMS analyses. The glassy inclusions that acted as Pb sinks and the microcrystalline SiO_2_ (+baddeleyite) resulting from annealing easily dissolve in HF, and the baddeleyite, once deprived of surrounding silica, “falls” apart from the well-recrystallized areas of zircon grains.

In principle, we did not anticipate that common lead, not structurally bounded to zircon lattice, would be so easily removed by percolating molten inclusions, leaving only radiogenic Pb in the structurally rebuilt parts of the zircon grain. Nevertheless, this fact might explain why high-*T* zircons, whether metamorphic^35−37^ or xenocrysts scavenged by hot mafic magmas^38−40^ rarely contain common lead and are either concordant or fit nicely in discordia lines. This fact suggests that the leaching mechanism described here might occur frequently in overheated zircons.

### 5.5 Opening Pathways for the Interaction of External Fluids with Zircon

The various transport pathways generated by annealing can connect the zircon surface with its interior, allowing entry to mobile external phases. In the annealed SAB50 and SAB51 zircons, there is textural and compositional evidence indicating that the formation of abundant low-*K* high-Si rhyolite melts required a significant contribution from the external silica sealant. The untreated zircons contain many feldspars but very few quartz inclusions. Nonetheless, the glass compositions of annealed zircons show high normative quartz, and the compositions lie in the primary phase field of silica minerals in the Qz-Ab-Or ternary, far from the quartz-feldspar cotectic valleys (Figure 11F). This implies that the melts were saturated in a silica mineral. Given the scarcity of quartz inclusions, the only silica-saturating phase could be the external silica sealant. Finally, the occurrence of rhyolite glass at textural sites that were devoid of felsic silicate minerals in the untreated grains, that is, the metamict porous bands with only some trace-element impurities in SAB51 zircons and the core–rim interfaces in SAB50 zircons, suggests transport on the scale of a single grain, as required for the entrance of an external mobile phase.

A contribution of the external silica sealant could be also significant for the generation of the calcic andesite to dacite melt in the annealed REG20 zircons, giving its high porosity (Figures 6 and 10), although its composition differs significantly from that of the melts saturated in a silica mineral.

**Figure 15:**
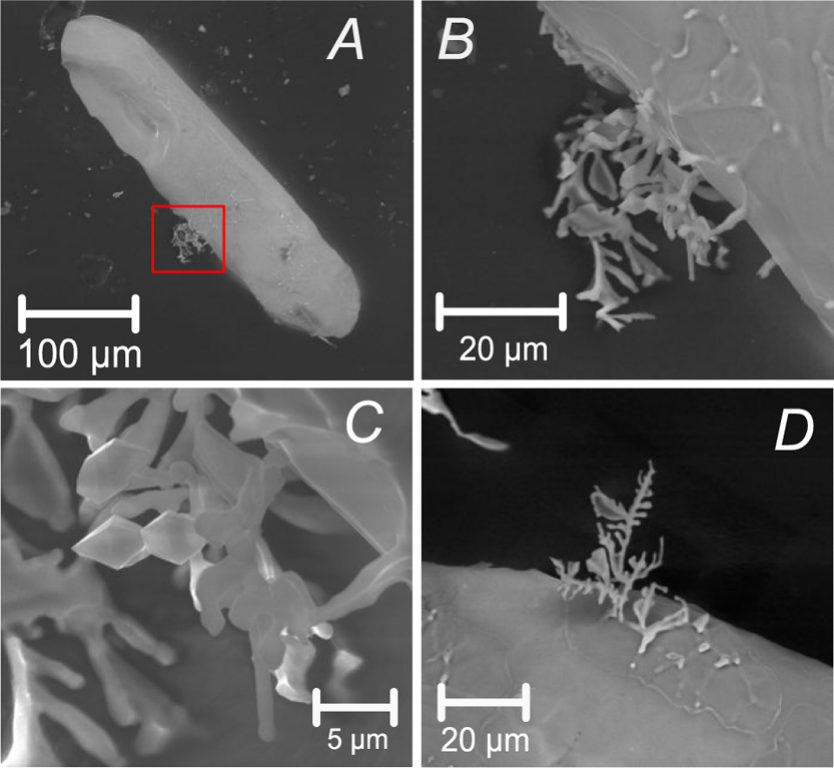
SE images of annealed zircon grains from tonalite SAB51 (A–C) and orthogneiss SAB50 (D). Note the formation of dendrites composed of tiny but euhedral zircon crystals at the zircon–cristobalite boundaries.

The reported open-system behavior not only involved the ingress of silica vapor but also the outflow of a mobile phase from the zircon interior. A key to understanding this is the observation of dendrites composed of tiny zircon crystals at the zircon–cristobalite boundaries (Figure 15). Since there is no evidence of zircon evaporation at the run temperatures, the appearance of tiny but euhedral zircon crystals suggests that they could have been generated in the presence of a melt released from the zircon interior.

### 5.6 Zircon–Baddeleyite Stability Relationships

The breakdown of unaltered pure zircon to SiO_2_ + ZrO_2_ occurs at a temperature close to 1675 °C (1676 ± 7;^41^ 1674 ± 7;^42^ and 1673 ± 10 °C,^43^). However, the presence of impurities and metamictization cause the appearance of baddeleyite at temperatures below 1600 °C (e.g., 1380–1480;^44^ 1477;^45^ 1285;^46^ 1400;^47^ and 1400 °C;^3^ see also refs (43) and (48)). Notably, baddeleyite has also been reported at even lower temperatures by recrystallization of highly metamict zircon (927;^49^ 852;^50^ 800–950;^51^ 600–900;^52^ and 900 °C,^53^).

In the annealing experiments presented in this work, baddeleyite forms by two main mechanisms: (i) recrystallization of metamict domains assisted by silica migration from the reaction site and (ii) incongruent dissolution of zircon into molten mineral inclusions of appropriate composition.

The first mechanism is responsible for generating tiny baddeleyite crystals in the porous bands of SAB51 zircons and of anhedral baddeleyite in porous domains of highly metamict REG20 zircons. The larger grain size and higher abundance of baddeleyite in the latter result from more extensive metamictization. In both cases, baddeleyite grains are surrounded by cathodoluminescent zircon produced by recrystallization of the metamict domains. It has been proposed that amorphous zircon recrystallizes via demixing of the metamict zircon into more stable oxides (e.g., refs (54) and (55); see ref (3) for further discussion). This implies the transient growth of a silicate mineral and baddeleyite and the subsequent elimination of the silica excess to preserve baddeleyite. The transport of excess silica to the outside is facilitated by the high porosity that the metamict domains already had and the secondary porosity that was created during zircon recrystallization, as indicated in the previous section.

The relatively coarser euhedral baddeleyite hosted in SAB50 and SAB51 zircons was likely produced by the second mechanism, that is, incongruent zircon dissolution. The textural relationships show that mineral inclusions can melt, reacting with the host zircon to produce baddeleyite. Most of them are enriched in fluorine, but calcic silicates can also give rise to the appearance of baddeleyite. Therefore, the compositional relationships suggest that zircon + baddeleyite can coexist with a large variety of melts ranging from ultrabasic fluorosilicate to fluorine-free andesite and dacite. Interestingly, the only compositional feature shared by all these melts is a high CaO content (>8 wt %). Hydrothermal experiments by Lewerentz et al.^52^ have shown baddeleyite formation after zircon when the molar amount of CaO in the fluid is close to or greater than that for silica. In the baddeleyite + zircon-saturated melts, the molar CaO/SiO_2_ ratio is very variable, ranging from 0.14 to 1.9, but it is always significantly higher than in baddeleyite-undersaturated melts (around 0.04). These data suggest that a high CaO abundance in the melt may favor baddeleyite saturation, although the compositional requirements are not as strict as in fluids.

## 6 Conclusions

Heat treatment of natural zircon grains from three different samples shows a series of phenomena that may find practical applications on one hand and, despite the high temperature conditions, may permit understanding the behavior of zircons during thermal shock related to magma hybridization and rock assimilation on the other hand.

Annealed zircons experience recrystallization of metamict domains, melting of polymineralic inclusions, formation of nanopores, and microcracking propagated by thermo-elastic stress accumulated at the interface between domains with different lattice orientations. The enhanced porosity permits melt migration through the crystal, leaching out impurities.

Annealed zircons that contained minute inclusions of rutile or any other Ti-bearing minerals increased their Ti concentrations in the lattice (SAB51 and SAB50 zircons). Zircons from a region with abundant W deposits (^21,22^) have high W concentrations. Upon annealing, minute W-mineral impurities also dissolved in the zircon lattice, causing an overall increase in W concentration in the zircon. In addition, a fraction of the released W was consumed in the formation of W-rich minerals, such as scheelite, because the solubility of W in zircon is limited.

Highly metamict zircons with elevated common Pb and radiogenic Pb loss, such as the REG20 ones, are impossible to date with SHRIMP. However, upon annealing at 1300 °C, they lost all their common Pb but little radiogenic Pb, producing well-fitted discordias with a meaningful upper intercept age. The requisite for losing almost all common Pb is the formation of the melt. We suggest that this is a way to date “undatable” zircons. We suggest that common Pb dwells in metallic or Pb oxide nanospheres, whereas radiogenic Pb remains attached to the zircon lattice.

During annealing, baddeleyite is formed at *T* considerably lower than the thermal decomposition temperature of pure zircons. We identified two main mechanisms: (i) recrystallization of metamict domains assisted by silica migration from the reaction site and (ii) incongruent dissolution of zircon into molten mineral inclusions with a high CaO/SiO_2_ ratio.

The formation of nanopores and microcracks enhances the intracrystalline melt mobility during the high *T* episode, leaching out mineral impurities and common lead. However, since the permeability of the zircon grain is increased by the creation of these open spaces, dry conditions should be maintained during cooling to prevent the entry of polluting fluids.

## Supporting Information Available

Microphotograph of zoned zircon grain from orthogneiss SAB50 under crossed polarized light, CL and SE images of annealed zircon grains from tonalite SAB51, and CL and SE images of annealed zircon grains from tonalite SAB50 (PDF)
LA-ICP-MS analyses of trace elements in zircon (XLSX)
SHRIMP data of untreated zircon (XLSX)
SHRIMP data of annealed zircon (XLSX)
