# Supplementary material for: Experimental Annealing of Zircon: Influence of Inclusions on Stability, Intracrystalline Melt Migration, Common Lead Leaching, and Permeability to Fluids
Source: ACS Earth Space Chem. 2022 Jan 11;6(2):288–307. doi: 10.1021/acsearthspacechem.1c00212 (PMC8862150; doi:10.1021/acsearthspacechem.1c00212)
Supplement: Supplementary file 1 — sp1c00212_si_001.pdf [file sp1c00212_si_001.pdf]

Experimental annealing of zircon: Influence of inclusions on stability, intracrystalline melt migration, common-lead leaching, and permeability to fluids

Irene Morales, José F. Molina, Aitor Cambeses, Pilar Montero and Fernando Bea*

Departamento de Mineralogía y Petrología, Facultad de Ciencias, Campus de Fuentenueva,

University of Granada, 18071-Granada, Spain

*Corresponding author: jfmolina@ugr.es; phone + 34 958246611.

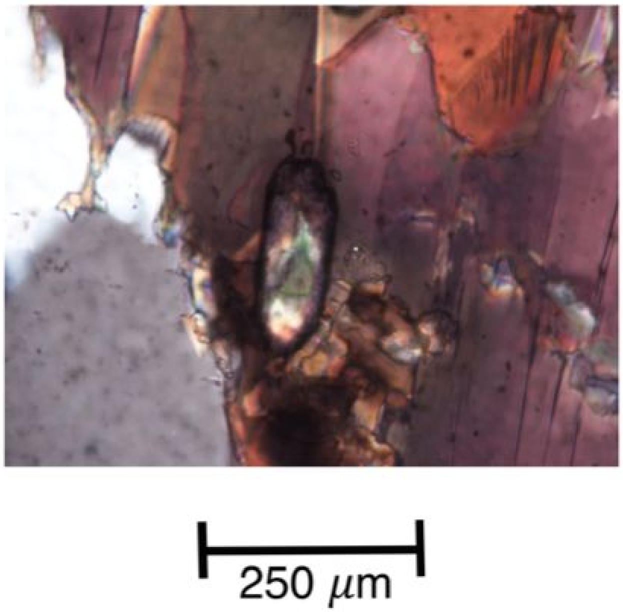

Figure S1. Microphotograph of zoned zircon grain from orthogneiss SAB50 under crossed polarized light showing a heterogeneous extinction what suggests that core and rims have a different lattice orientation.

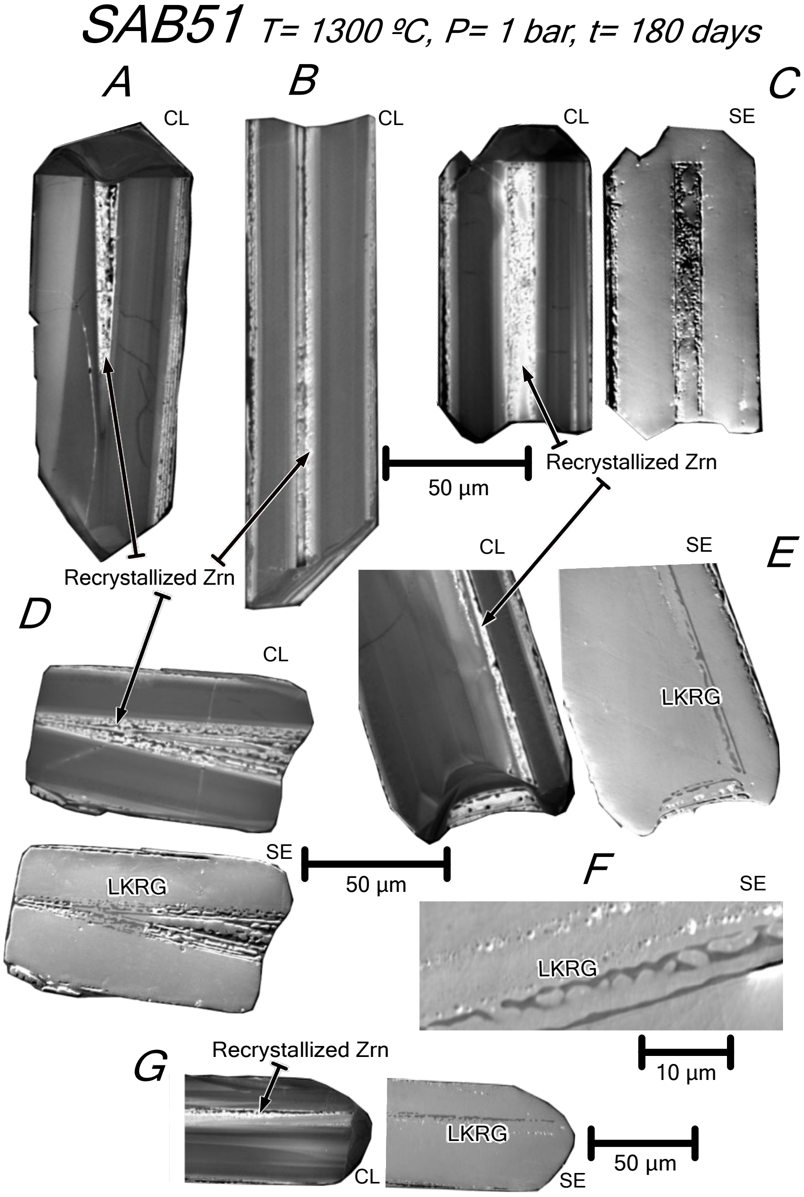

Figure S2. Cathodoluminescence (CL) and secondary electron (SE) images of annealed zircon grains from tonalite SAB51. **A**, **B** and **C)** Zircons showing that the bands parallel to prismatic and pyramidal faces become luminescent after heat treatment suggesting recrystallization. **D**, **E** and **G)** Zircons with luminescent, recrystallized, bands that hots inclusion of low-K rhyolitic glass; the micropores of the bands were filled with low-K rhyolitic melt during the heat treatment. **F)** A

zircon detail of band with inclusion of low-K rhyolite glass. Abbreviations: LKRG = low-K rhyolite glass.

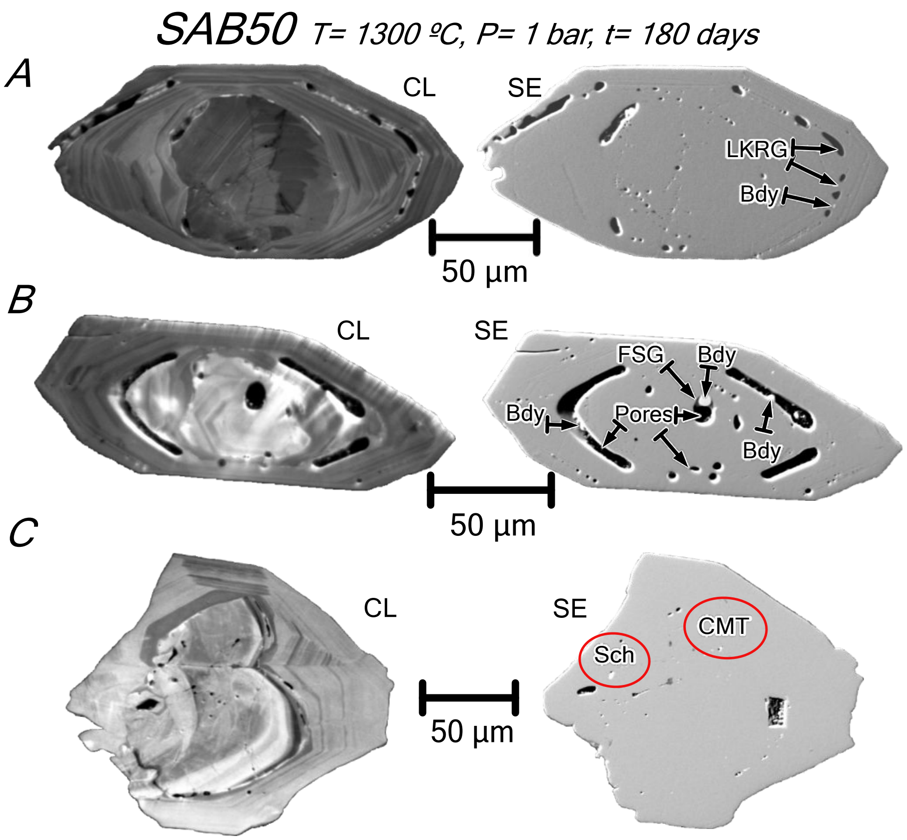

Figure S3. Cathodoluminescence (CL) and secondary electron (SE) images of annealed zircon grains from orthogneiss SAB50. **A)** Zircon hosting single-phase inclusions of baddeleyite and low-K rhyolite glass lying along the core-rim interface. **B)** Zircon with oscillatory zoning and a shell rimming a luminescent inherited core hosting a tear-drop shaped multi-phase inclusion of fluorosilicate glass + euhedral baddeleyite with a micropore and baddeleyite inclusions lying along elongated micropores with rounded tips developed at the core-rim interface. **C)** Zircon

with a core truncated by a rim that hosts monomineralic inclusions of scheelite and Ca-Mg tungstate, one located at the core-rim interface and the other in the core (red ovals). Mineral abbreviations after Whitney and Evans [8]. Other abbreviations: CSG = calcic silicate glass; FSG = fluorosilicate glass; KRG = potassic rhyolite glass; LKRG = low-K rhyolite glass; SH = SHRIMP analysis spot; CMT = Ca-Mg tungstate.
